# Agreement between self-reported and measured weight, height, and derived BMI by educational attainment across racial and ethnic groups of US women

**DOI:** 10.1038/s41366-025-01784-8

**Published:** 2025-04-14

**Authors:** Symielle A. Gaston, Karla N. Kendrick, Bethany T. Ogbenna, Dale P. Sandler, Fatima Cody Stanford, Chandra L. Jackson

**Affiliations:** 1 Epidemiology Branch, National Institute of Environmental Health Sciences, National Institutes of Health, Research Triangle Park, NC USA; 2 Winchester Hospital Weight Management Center, Beth Israel Lahey Health, Woburn, MA USA; 3 Massachusetts General Hospital, MGH Weight Center, Department of Medicine-Division of Endocrinology-Neuroendocrine, Department of Pediatrics-Division of Endocrinology, Boston, MA USA; 4 Division of Intramural Research, National Institute on Minority Health and Health Disparities, National Institutes of Health, Bethesda, MD USA

**Keywords:** Risk factors, Epidemiology

## Abstract

**Objective:**

Underreporting of weight and overreporting of height is consistently shown among women, thereby reducing accuracy of estimation of body mass index—and thus obesity—in epidemiologic studies that rely on self-reported data. Additionally, misreporting has been shown to differ by socioeconomic status and race and ethnicity, which can result in differential misclassification and bias that can obfuscate associations with obesity across groups in multiethnic and socioeconomically varying populations. Therefore, we sought to assess agreement between self-reported and objectively measured weight, height, and derived body mass index (BMI) across levels of educational attainment within racial and ethnic groups in a population-based cohort of US women.

**Methods:**

Among self-identified White, Black, and Latina women enrolled in the Sister Study (2003–2009), we assessed mean differences in self-reported vs. objectively measured weight, height, and derived BMI. Using adjusted linear and multinomial logistic regression, we compared measurement error among participants reporting some college/vocational school or ≥college vs. ≤high school. We assessed BMI agreement using Bland-Altman plots and weighted kappa (*k*) statistics.

**Results:**

Among 18,638 participants (age: mean ± standard deviation = 56 ± 9.0 years), 84% identified as White, 10% Black, and 5% Latina. Approximately half (49%) attained a college education. Weight and height were generally underreported. Higher underreporting of weight among participants with ≥college vs. ≤high school was of larger magnitude among Black and Latina vs. White participants. Bland-Altman results revealed that agreement in continuous BMI was good among White participants but generally fair among Black and Latina participants. Categorical BMI agreement was consistently high with minor variation by race and ethnicity and educational attainment (weighted *k* range = 0.92–0.93).

**Conclusions:**

Despite higher measurement error in weight among Black and Latina participants with ≥college education, self-reported and objectively measured BMI categories showed high agreement across groups. Results support the utility of self-reported data that reliably estimate BMI category across socioeconomic, racial, and ethnic groups in this cohort.

## Introduction

Obesity is characterized by excess weight, potentially accompanied by detectable hormonal and metabolic disturbances, that may eventually lead to a host of adverse health outcomes, such as type 2 diabetes, cardiovascular disease, and certain cancers (e.g., colon) as well as premature mortality [1–4]. Body mass index (BMI) is carefully tracked in clinical settings and for epidemiological purposes as an essential measure to screen for obesity ( ≥ 30 kg/m^2^) and its relationship to subsequent disease and mortality risk. However, population-level data to determine associations with and temporal trends in obesity often rely on self-reported weight and height. Still, self-reports can lead to inaccurate estimates of obesity prevalence [5–9].

Previous studies among women have demonstrated that self-reported weight is consistently underestimated compared to objectively measured weight, while height is generally though inconsistently overestimated [6–16]. Moreover, degrees of misreporting of weight and height vary by myriad individual-level characteristics such as objective weight, objective height, race, ethnicity, and educational attainment [13, 15, 17–19]. For example, in a prior analysis among early enrollees in the Sister Study cohort, weight underreporting was higher among Black/African American (hereafter Black) compared to non-Hispanic White (hereafter White) women, women with less than a Bachelor’s vs. a Bachelor’s degree, and women with overweight/obesity vs. recommended weight [20]. Relatedly, additional research suggests that unlike in other racial and ethnic groups, relationships between higher socioeconomic status and lower obesity status among Black women are observed when BMI is subjectively but not objectively measured [11]. Potential reasons include differential misreporting of weight by educational attainment, where women with higher educational attainment are more likely to underreport weight than women with lower educational attainment [17, 19]. Therefore, bias in associations between BMI and health outcomes could vary depending on the modality (e.g., self-report, objectively measured) employed among this group.

Given previously observed reporting differences by race-ethnicity and socioeconomic status, separately but not in combination [10–13, 17–25], complex interactions by these social characteristics may result in variation in the accuracy of self-reported anthropometrics in distinct populations, potentially resulting in differential misclassification of obesity within racially and ethnically heterogenous samples. Implications of differential misclassification and underestimation of the true prevalence of obesity among women, racially and ethnically minoritized groups, and individuals with lower socioeconomic position who have the highest burdens of obesity in the US [26–28] is of public health importance [10, 14]. For instance, consequences may include misallocation of resources and failure to prevent and address potentially adverse consequences of obesity among socially disadvantaged groups. Within extensive, multiethnic studies that rely on self-reported data, implications include potential measurement error bias when determining obesity status, which can then obfuscate obesity-disease associations across racial and ethnic groups. To determine this possibility and fill a research gap using a large, ongoing cohort study that repeatedly collects self-reported anthropometric data, the objectives of the current study were to determine whether measurement error of self-reported vs. examiner-/objectively measured anthropometric measures (i.e., weight, height, derived BMI) varies by educational attainment among Sister Study participants, overall and within racial and ethnic groups. We hypothesized that measurement error of weight and derived BMI varies differentially by educational attainment within racial and ethnic groups: the magnitude of measurement error will be highest among women with the highest educational attainment within Black women. Specifically, we hypothesized that Black women with a college degree or greater are more likely to underreport their weight and thus have underestimated BMI compared to Black women with a high school diploma or less. Lastly, we hypothesize that there will be no differences in measurement error of height by race and ethnicity: irrespective of race and ethnicity, women with higher (college/some college) vs. lower (≤high school) educational attainment will be less likely to misreport height.

## Materials and methods

### The Sister Study

The Sister Study is a prospective cohort study of environmental, behavioral, and lifestyle correlates of breast cancer. The Sister Study protocol is described in detail elsewhere [29]. Briefly, women were recruited using multiple modalities (e.g., clinic visits, radio advertisements, word-of-mouth). To meet eligibility criteria, US women had to be between the ages of 35 and 74 years and have a sister previously diagnosed with breast cancer but be breast cancer free themselves at the time of baseline data collection (2003-2009). If eligible women agreed to enroll, they were mailed study kits and were contacted to (1) confirm receipt, (2) review the materials provided in the kit, and (3) learn what to expect as a study participant. During baseline data collection, participants provided data through two-part computer-assisted telephone interviews (CATIs) and self-administered questionnaires, and trained clinical personnel collected anthropometric measures during an in-home visit. All participants provided informed consent. The National Institute of Environmental Health Sciences Institutional Review Board and the Copernicus Group approved Sister Study protocol. Approval for the current study of de-identified secondary data (Release 10.1) was waived. This study follows the Guidelines for Reporting Reliability and Agreement Studies (GRRAS) [30].

### Analytic sample

Participants who did not meet inclusion criteria (detailed in Supplementary Fig. 1), including participants who did not complete the two-part CATI prior to the in-home visit, were removed from the analytic sample in a stepwise manner. The final analytic sample comprised 18,368 White, Black, and Hispanic/Latina (hereafter Latina) women. Compared to women in the analytic sample, ineligible/excluded women were more likely to: be younger, not self-identify as White, have a higher socioeconomic status, both report and objectively have a lower weight, more accurately report weight and height, report more weight fluctuations of 20 pounds/9.07 kilograms (kg) or more over the life course, have an objectively measured BMI within the recommended range (18.5–24.9 kg/m^2^), and report less poor health (Supplementary Table 1). Additionally, 45% of ineligible participants completed the home visit/exam >30 days prior to the CATI. Lastly, participants who completed the home visit/exam either prior to or after the CATI and met the other inclusion criteria were also included in a secondary analysis of 46,618 participants in which we sought to both compare results of the main analysis and investigate agreement in the wider cohort.

### Measurements

#### Socioeconomic status

We used self-reported educational attainment, a social determinant of health, as a measure of socioeconomic status, particularly because education can determine income and other socioeconomic factors (e.g., occupation) that influence health [31]. Categories included ≤high school, some college or vocational school (hereafter some college), and ≥college (Bachelor’s degree or higher).

#### Anthropometrics

During the in-home visit, trained examiners collected weight, height, other anthropometrics, and blood pressure measurements following standardized protocols [20, 29]. Briefly, self-identified female examiners used digital self-calibrating scales to measure weight without shoes and a metal tape measure to measure height. Each measurement was taken three times and averaged. During Part Two of the CATI, participants self-reported their current weight and height as well as their weight and height histories during childhood and adulthood. Weight was rounded to the nearest tenth of a pound (lb), and height was rounded to the nearest quarter of an inch. These values were then converted to kilograms (kg) and centimeters (cm). We then calculated BMI as kg/meters^2^. BMI (kg/m^2^) was considered continuously and categorically using standard categories (described in Table 1): underweight, recommended weight, overweight, Class I obesity, Class II obesity, and Class III obesity [32].

**Table 1:** Study population characteristics at enrollment by educational attainment across race and ethnicity, Sister Study (2003–2009), N = 18,368.

Characteristic	White, n = 15,502 (84%)				Black, n = 1857 (10%)				Latina, n = 1009 (5%)			
	Overall, n = 15,502^a^ (100%)	≤HS, n = 2591^a^ (17%)	Some College, n = 5323^a^ (34%)	≥College, n = 7588^a^ (49%)	Overall, n = 1857^a^ (100%)	≤HS, n = 159^a^ (9%)	Some College, n = 679^a^ (37%)	≥College, n = 1019^a^ (55%)	Overall, n = 1009^a^ (100%)	≤HS, n = 219^a^ (22%)	Some College, n = 363^a^ (36%)	≥College, n = 427^a^ (42%)
Age, years	56.3 (8.93)	57.6 (9.00)	56.2 (8.69)	55.9 (9.04)	52.7 (8.13)	53.5 (8.22)	52.6 (8.30)	52.6 (8.00)	52.5 (9.17)	54.4 (9.10)	52.6 (8.97)	51.5 (9.25)
Age category												
35–64 years	79	74	79	80	92	91	91	92	89	86	90	90
65+ years	21	26	21	20	8	9	9	8	11	14	10	10
Annual household income												
<$20,000	4	7	4	2	6	17	11	1	16	39	15	4
$20,000–<$50,000	21	35	24	13	26	43	35	17	30	33	34	25
$50,000–$99,999	42	43	45	39	42	30	37	47	32	21	34	35
≥$100,000	34	15	26	46	27	10	17	35	22	7	16	36
Marital status												
Married/living as married	77	80	77	76	51	45	48	54	69	68	69	70
Single/never married	4	2	3	6	15	14	14	16	8	8	5	10
Divorced/separated/widowed	18	18	20	17	34	40	38	30	23	25	26	20
Self-reported weight (kg)	73.4 (16.28)	75.5 (17.09)	75.2 (16.53)	71.4 (15.57)	83.1 (18.73)	86.2 (19.16)	84.8 (19.78)	81.5 (17.77)	71.7 (15.40)	73.1 (14.76)	72.3 (16.22)	70.4 (14.94)
Objectively-measured weight (kg)	74.3 (16.82)	76.3 (17.58)	76.1 (17.06)	72.3 (16.13)	84.5 (19.38)	86.9 (19.27)	86.1 (20.35)	83.1 (18.62)	72.4 (16.09)	73.1 (15.47)	73.2 (16.93)	71.3 (15.65)
Self-reported vs. objectively measured weight (lbs/kg)												
Under-report by ≤7 lbs/3.2 kg	16	16	17	16	25	23	25	26	17	12	20	17
Under-report by 4 lbs/1.8 kg–<7 lbs/3.2 kg	16	16	16	16	14	14	14	14	11	9	10	13
Within 4 lbs/1.8 kg	55	52	54	56	45	42	44	47	56	58	53	57
Over-report by ≥4 lbs/1.8 kg	13	16	13	12	16	20	18	13	16	21	17	14
Self-reported vs. objectively measured weight (%)												
Under-report ≤5%	12	11	12	12	18	17	17	18	13	10	15	14
Within 5%	85	85	84	85	78	74	77	79	81	82	79	82
Over-report ≥5%	3	4	3	3	4	9	5	3	6	8	6	4
Self-reported Height (cm)	164.2 (6.51)	163.2 (6.44)	164.0 (6.41)	164.7 (6.55)	164.1 (6.93)	163.0 (7.13)	163.5 (6.79)	164.6 (6.94)	160.4 (6.23)	159.9 (5.76)	159.8 (6.65)	161.2 (6.03)
Objectively measured Height (cm)	164.4 (6.35)	163.4 (6.22)	164.2 (6.25)	164.9 (6.41)	164.5 (6.68)	163.3 (6.59)	164.1 (6.62)	165.1 (6.68)	160.5 (6.37)	159.6 (6.15)	160.2 (6.76)	161.2 (6.06)
Self-reported vs. objectively measured height (in/cm)												
Underreport by ≥1 in/2.54 cm	14	16	15	14	21	18	24	20	18	17	20	16
Within 1 in/2.54 cm	75	72	74	76	68	65	64	70	66	62	65	69
Overreport by ≥1 in/2.54 cm	11	12	11	10	11	17	11	10	16	21	15	15
Self-reported BMI (kg/m^2^)	27.3 (5.82)	28.4 (6.14)	28.0 (5.89)	26.4 (5.50)	30.9 (6.67)	32.5 (6.96)	31.8 (7.03)	30.1 (6.27)	27.9 (5.63)	28.6 (5.35)	28.3 (6.06)	27.1 (5.30)
Objectively Measured BMI (kg/m^2^)	27.5 (6.02)	28.6 (6.33)	28.3 (6.09)	26.6 (5.72)	31.3 (6.91)	32.7 (7.01)	32.0 (7.20)	30.6 (6.61)	28.2 (6.00)	28.7 (5.72)	28.6 (6.48)	27.5 (5.64)
Self-reported BMI category												
Underweight	1	0	1	1	0	0	0	0	0	0	1	0
Recommended	40	32	34	47	17	11	13	21	34	26	33	40
Overweight	32	34	34	30	34	31	33	34	36	38	35	36
Class I Obesity	17	20	19	14	26	26	26	26	19	27	18	15
Class II Obesity	6	8	8	5	13	16	15	12	6	5	6	5
Class III Obesity	4	5	5	3	10	16	12	8	5	4	7	3
Objectively measured BMI category												
Underweight	1	1	1	1	0	0	0	0	1	0	1	0
Recommended	38	30	33	45	17	10	13	20	33	25	31	38
Overweight	32	34	33	30	31	30	29	32	37	43	36	35
Class I Obesity	17	19	19	15	26	28	28	25	18	20	18	16
Class II Obesity	7	9	9	6	14	16	16	13	6	8	6	6
Class III Obesity	4	6	5	3	11	17	13	9	5	4	9	4
Weight fluctuations (by ±20 lbs/ 9.1 kg) over the life course												
Never	53	48	49	57	49	52	46	50	64	70	61	64
1 to 2 times	25	28	26	24	27	23	28	28	22	21	26	19
3+ times	22	24	25	19	24	25	26	22	14	9	13	17
Fair/Poor general health	5	8	6	4	10	14	15	7	17	32	18	9
Antidepressant use	22	22	24	21	11	8	14	10	14	14	15	12
Time between exam and CATI												
Days	39.9 (79.6)	35.8 (56.5)	40.2 (75.2)	41.0 (88.8)	56.6 (83.2)	50.7 (65.8)	58.1 (91.8)	56.6 (79.5)	56.8 (88.4)	66.8 (135)	49.9 (62.2)	57.5 (76.1)
EX & SR within 30 days	67	66	66	68	52	53	52	51	48	46	52	46
EX > 30 days after SR	33	34	34	32	48	47	48	49	52	54	48	54

Percentages may not sum to 100 due to rounding. All characteristics varied by educational attainment within each racial and ethnic group (two-sided p-value for Pearson’s Chi-squared test <0.05), except the following: age among Black women; marital status among Latina women; self-reported weight among Latina women; objectively measured weight among Latina women; differences in self-reported and objectively measured weight (kg) among Black and Latina women; differences in self-reported and objectively measured weight (%) among White and Latina women; differences in self-reported and objectively measured height (cm) among Latina women; weight fluctuations over the life course among Black women; antidepressant use among Black and Latina women; and time between exam and CATI.

*HS* high school, *lbs* pounds, *kg* kilograms, *cm* centimeters, *in* inches, *BMI* body mass index, *CATI* computer-assisted telephone interview, *EX* examiner/objectively measured, *SR* self-reported.

^a^Mean (SD); %.

#### Concordance measures

Consistent with a prior study of early enrollees in the Sister Study cohort [20], self-reported vs. objectively measured weight concordance measures included categorical absolute differences (under-report by ≥7 lbs/3.18 kg, under-report by 4–<7 lbs/1.81–<3.18 kg, reporting within 4 lbs/1.81 kg [reference], over-report by ≥4 lbs/1.81 kg) and categorical percent differences (under-report by ≥5%, report within 5% [reference], over-report by ≥5%). Also consistent with the prior analysis, self-reported vs. objectively measured height categories included under-report by ≥1 inch/2.54 cm, report within 1 inch/2.54 cm (reference), over-report by ≥1 inch/2.54 cm.

#### Potential confounders

All potential confounders were self-reported (unless otherwise noted) at the baseline CATI and were chosen a priori based on prior literature [20]. As listed in Table 1, potential confounders included self-identified race and ethnicity (White [non-Hispanic], Black [non-Hispanic], Latina; in overall models), continuous age, marital status, objectively measured height (cm), BMI category (derived from objective measures), frequency of weight fluctuations (gain and loss) of ≥20 lbs/9.07 kg over the life course, perceived general health status, current antidepressant use, and time between self-report of weight/height and the home visit/exam.

#### Potential modifiers

Race along with ethnicity and educational attainment were also considered potential modifiers.

### Statistical analysis

Study population characteristics were described as means ± standard deviations (SD) for continuous variables and as proportions for categorical variables in the total population and within races/ethnicities, stratified by educational attainment. There were influential outliers for weight and derived BMI, we therefore winsorized extreme values by replacing their true values with the 1^st^ and 99^th^ percentile value.

Bland-Altman plots that included means of differences between self-reported and objective measurements as well as upper and lower limits of agreement (LoA) were used to visualize agreement [33–38]. As described in prior literature [33, 34, 36, 38], Bland-Altman plots allow for visualization of bias and the corresponding LoA, which show where 95% of the differences between measures fall. However, the Bland-Altman plot itself does not provide information on whether the differences between measures are acceptable (i.e., whether there is good agreement), and acceptable limits must be determined a priori [33, 34, 36, 38]. To determine the acceptability of the agreement between self-reports and objective measures, we compared the widths of the LoA to the SDs of the means of objectively measured values, which is consistent with prior literature [39, 40]. Since the LoA and SDs are two measures of variation, direct comparisons of the two indicate whether the variability in measurement error (i.e., width of the LoA) introduces additional variability beyond the expected variability in the objective measure. Consistent with prior literature [39, 40], ‘good’ agreement was defined as widths of LoA that are within one SD, ‘fair’ agreement was defined as widths of LoA that are greater than one but within two SDs, and ‘poor’ agreement was defined as widths of LoA that were within three SDs. We also used paired samples t-tests to estimate mean differences and 95% confidence intervals between objectively measured and self-reported weight, height, and continuous BMI in the overall population, within educational attainment categories, and within combined race and ethnicity and educational attainment categories.

To address potential bias due to differences between the analytic sample and excluded participants, we estimated stabilized inverse probability weights (IPWs) for missingness, and these weights were applied to models. Weighted linear regressions estimated educational attainment categories (i.e., some college or ≥college vs. ≤high school) in relation to mean differences (MDs) and 95% confidence intervals (CIs)) in measurement errors of continuous weight, height, and BMI, overall and by race and ethnicity. Using weighted multinomial logistic regression, we estimated odds ratios (ORs) and 95% CIs for the categorical concordance measures for weight and height (e.g., under-report height by ≥1 inch/2.54 cm vs. within 1 inch/2.54 cm) among the total population and by race and ethnicity. For consistency with the prior study of early enrollees in the Sister Study [20], all models were adjusted for age, marital status, BMI category, weight fluctuations, perceived general health, anti-depressant use, and time between the in-home exam and CATI; overall models were additionally adjusted for race and ethnicity. We additionally adjusted for height given prior evidence of differences in reporting by height [10]. Lastly, we assessed proportions, the percentage agreement, the kappa (*k*), the weighted *k*, sensitivity, and specificity for BMI categories based on self-reports vs. objective measurements, overall, by educational attainment, and by educational attainment within racial and ethnic groups. Weighted *k* statistics were interpreted due to the ordinal nature of BMI categories as follows: no agreement (*k* = 0–0.20); minimal to weak agreement (*k* = 0.21–0.59); moderate to strong agreement (*k* = 0.60–0.90); almost perfect agreement (*k* ≥ 0.90) [41]. Sensitivity and specificity calculations were based on obesity vs. non-obesity status.

As secondary analyses, we repeated Bland Altman analyses and analyses of agreement between categorical BMI measures while including participants who completed self-reports after exams (n = 46,618). We did not perform multiple comparison correction since we tested a priori hypotheses [42]. All analyses were performed using RStudio, 2022.02.0 Build 443 with the packages listed in Supplementary Table 2, and a two-sided p-value of 0.05 determined statistical significance.

## Results

### Study population characteristics

Among 18,368 participants with a mean ± standard deviation (SD) age of 56 ± 9.0 years, 84% identified as White, 10% as Black, and 5% as Latina (Supplementary Table 1). Almost half of participants (49%) completed college. Across races and ethnicities, most characteristics varied by educational attainment (Table 1). Means of objectively measured weight were higher than means of self-reported weight. While mean weight was similar among White and Latina women who attained ≤high school and some college, it was lower among White and Latina women who attained ≥college. Self-reported and objectively measured weight decreased with increasing educational attainment among Black women (e.g., objectively measured mean ± SD weight: ≤high school = 87 ± 19 kg, some college = 86 ± 20 kg, ≥college = 83 ± 19 kg). Most participants reported weight within 5% of examiner measurements; however, the proportion within 5% varied by race and ethnicity (White 85%, Black 78%, Latina 81%). Self-reported and objectively measured heights were similar. Obesity prevalence based on self-reported and examiner measurements was 27% and 28% among White women, 49% and 51% among Black women, and 30% and 29% among Latina women. Whether self-reported or measured, obesity prevalence decreased as educational attainment increased. Most White (53%) and Latina (64%) participants reported no weight fluctuations (by +/- 20 lbs/9.1 kg) over the life course; however, less than half (49%) of Black participants reported no weight fluctuations over the life course. Most participants did not report fair/poor general health. The average time between CATI completion and examination was 42 ± 81 days, overall. While most White participants (67%) had their examination within 30 days of the CATI interview, approximately half of Black participants (52%) and Latina participants (48%) had their examination within 30 days. Categorical time between the exam and CATI did not vary by educational attainment within racial and ethnic groups.

### Measurement error overall, by race and ethnicity, and by educational attainment

Bland-Altman plots are included in Supplementary Materials (Supplementary Figs. 2–4) and are described in Table 2. Generally, White, Black and Latina participants underreported weight, height, and resultantly had lower derived self-reported vs. objectively measured BMI. Limits of agreement were relatively wide, particularly among Black and Latina compared to White participants. However, across racial and ethnic groups as well as educational attainment categories, widths of the LoA for weight were less than the SDs of objective weight measurements (see Table 3 for the values), showing good agreement. Across racial and ethnic groups as well as educational attainment categories, agreement in height was fair as demonstrated by the widths of the LoA being within 2 SDs of objective height measurements. While widths of LoA were within one SD of BMI based on objective measures among White participants across educational attainment categories—suggesting good agreement, widths of LoA were within two SDs among Black participants who completed ≤high school and ≥college as well as among Latina participants across educational attainment categories, suggesting fair agreement.

**Table 2:** Bland-Altman plot statistics: bias (mean of differences between self-report and objective measures) and limits of agreement by educational attainment within racial and ethnic groups, Sister Study (2003–2009), N = 18,368.

	Mean of differences	SD of differences	(LLoA,	ULoA)	Mean of differences	SD of differences	(LLoA,	ULoA)	Mean of differences	SD of differences	(LLoA,	ULoA)
	White				Black				Latina			
Weight (kg)												
≤High School	**−0.77**	**2.97**	**(−6.59,**	**5.05)**	**−0.82**	**4.27**	**(−9.20,**	**7.56)**	**−0.07**	**3.35**	**(−6.64,**	**6.49)**
Some College	**−0.92**	**3.01**	**(−6.82,**	**4.99)**	**−1.26**	**3.96**	**(−9.01,**	**6.50)**	**−0.95**	**3.45**	**(−7.70,**	**5.81)**
≥College	**−0.93**	**2.71**	**(−6.24,**	**4.38)**	**−1.61**	**3.95**	**(−9.36,**	**6.14)**	**−0.86**	**3.01**	**(−6.75,**	**5.04)**
Height (cm)												
≤High School	*−0.17*	*2.26*	*(−4.59*,	*4.25)*	*−0.25*	*3.08*	*(−6.29*,	*5.80)*	*0.26*	*3.69*	*(−6.98*,	*7.50)*
Some College	*−0.20*	*2.22*	*(−4.55*,	*4.15)*	*−0.53*	*2.96*	*(−6.33*,	*5.26)*	*−0.34*	*2.91*	*(−6.04*,	*5.35)*
≥College	*−0.19*	*1.99*	*(−4.09*,	*3.71)*	*−0.41*	*2.64*	*(−5.59*,	*4.77)*	*−0.04*	*2.48*	*(−4.89*,	*4.81)*
BMI (kg/m^2^)												
≤High School	**−0.22**	**1.36**	**(−2.88,**	**2.44)**	*−0.23*	*2.05*	*(−4.25*,	*3.79)*	*−0.13*	*1.89*	*(−3.82*,	*3.57)*
Some College	**−0.27**	**1.37**	**(−2.95,**	**2.40)**	**−0.28**	**1.70**	**(−3.61,**	**3.05)**	*−0.28*	*1.62*	*(−3.45*,	*2.89)*
≥College	**−0.28**	**1.19**	**(−2.61,**	**2.05)**	*−0.45*	*1.71*	*(−3.79*,	*2.90)*	*−0.32*	*1.45*	*(−3.17*,	*2.53)*

An estimated 95% of differences are within the limits of agreement. Bolded values indicate ‘good’ agreement, defined as a width of the limits of agreement that is within one standard deviation of the objectively measured mean value. Italicized values indicate ‘fair’ agreement, defined as a width of the limits of agreement that equals greater than one but less than or equal to two standard deviations of the objectively measured mean value.

*SD* standard deviation, *LLoA* lower limit of agreement, *ULoA* upper limit of agreement.

**Table 3:** Mean differences between self-reported and objectively/examiner measured weight, height, and body mass index, overall and by educational attainment within the overall population and within racial and ethnic groups, Sister Study (2003–2009), N = 18,368.

	Overall							≤ High School							Some College							≥ College						
	Self-reported		Objectively measured		MD	(95% CI)		Self-reported		Objectively measured		MD	(95% CI)		Self-reported		Objectively measured		MD	(95% CI)		Self-reported		Objectively measured		MD	(95% CI)	
	Mean	(SD)	Mean	(SD)				Mean	(SD)	Mean	(SD)				Mean	(SD)	Mean	(SD)				Mean	(SD)	Mean	(SD)			
Weight (kg)																												
Overall	74.2	(16.3)	75.1	(16.9)	**−0.94**	**(−0.99,**	**−0.90)**	75.8	(16.7)	76.5	(17.2)	**−0.72**	**(−0.83,**	**−0.61)**	76.0	(16.7)	76.9	(17.2)	**−0.96**	**(−1.03,**	**−0.88)**	72.4	(15.7)	73.4	(16.4)	**−1.00**	**(−1.06,**	**−0.94)**
White	73.3	(15.9)	74.2	(16.5)	**−0.90**	**(−0.95,**	**−0.85)**	75.4	(16.5)	76.2	(17.0)	**−0.77**	**(−0.89,**	**−0.66)**	75.2	(16.2)	76.1	(16.8)	**−0.92**	**(−1.00,**	**−0.84)**	71.3	(15.2)	72.3	(15.8)	**−0.93**	**(−0.99,**	**−0.87)**
Black	82.8	(17.7)	84.2	(18.4)	**−1.41**	**(−1.59,**	**−1.23)**	85.9	(18.5)	86.7	(18.9)	**−0.82**	**(−1.49,**	**−0.15)**	84.3	(18.3)	85.6	(18.8)	**−1.26**	**(−1.55,**	**−0.96)**	81.2	(17.1)	82.9	(17.9)	**−1.61**	**(−1.85,**	**−1.37)**
Latina	71.6	(15.1)	72.3	(15.7)	**−0.72**	**(−0.92,**	**−0.52)**	73.1	(14.7)	73.2	(15.3)	−0.07	(−0.52,	0.37)	72.2	(15.7)	73.1	(16.5)	**−0.95**	**(−1.30,**	**−0.59)**	70.4	(14.6)	71.3	(15.2)	**−0.86**	**(−1.14,**	**−0.57)**
Height (cm)																												
Overall	164.0	(6.6)	164.2	(6.4)	**−0.21**	**(−0.24,**	**−0.18)**	163.0	(6.5)	163.1	(6.3)	**−0.14**	**(−0.23,**	**−0.05)**	163.7	(6.5)	164.0	(6.4)	**−0.24**	**(−0.30,**	**−0.18)**	164.5	(6.6)	164.7	(6.5)	**−0.21**	**(−0.25,**	**−0.16)**
White	164.2	(6.5)	164.4	(6.3)	**−0.19**	**(−0.22,**	**−0.16)**	163.2	(6.4)	163.4	(6.2)	**−0.17**	**(−0.26,**	**−0.08)**	164.0	(6.4)	164.2	(6.3)	**−0.20**	**(−0.26,**	**−0.14)**	164.7	(6.6)	164.9	(6.4)	**−0.19**	**(−0.23,**	**−0.14)**
Black	164.1	(6.9)	164.5	(6.7)	**−0.44**	**(−0.57,**	**−0.31)**	163.0	(7.1)	163.3	(6.6)	−0.25	(−0.73,	0.24)	163.5	(6.8)	164.1	(6.6)	**−0.53**	**(−0.76,**	**−0.31)**	164.6	(6.9)	165.1	(6.7)	**−0.41**	**(−0.57,**	**−0.25)**
Latina	160.4	(6.2)	160.5	(6.4)	−0.08	(−0.27,	0.10)	159.9	(5.8)	159.6	(6.2)	0.26	(−0.23,	0.75)	159.8	(6.7)	160.2	(6.8)	**−0.34**	**(−0.64,**	**−0.04)**	161.2	(6.0)	161.2	(6.1)	−0.04	(−0.27,	0.20)
BMI (kg/m^2^)																												
Overall	27.6	(5.9)	27.9	(6.1)	**−0.28**	**(−0.30,**	**−0.26)**	28.6	(6.0)	28.8	(6.2)	**−0.21**	**(−0.26,**	**−0.16)**	28.4	(6.0)	28.7	(6.2)	**−0.27**	**(−0.31,**	**−0.24)**	26.8	(5.6)	27.1	(5.8)	**−0.30**	**(−0.33,**	**−0.28)**
White	27.2	(5.7)	27.5	(5.9)	**−0.27**	**(−0.29,**	**−0.25)**	28.4	(6.0)	28.6	(6.1)	**−0.22**	**(−0.27,**	**−0.16)**	28.0	(5.8)	28.3	(6.0)	**−0.27**	**(−0.31,**	**−0.24)**	26.3	(5.4)	26.6	(5.6)	**−0.28**	**(−0.31,**	**−0.26)**
Black	30.8	(6.3)	31.2	(6.5)	**−0.37**	**(−0.45,**	**−0.29)**	32.4	(6.7)	32.6	(6.9)	−0.23	(−0.55,	0.09)	31.6	(6.5)	31.9	(6.7)	**−0.28**	**(−0.41,**	**−0.15)**	30.0	(6.0)	30.5	(6.3)	**−0.45**	**(−0.55,**	**−0.34)**
Latina	27.9	(5.5)	28.1	(5.8)	**−0.26**	**(−0.36,**	**−0.17)**	28.6	(5.2)	28.7	(5.5)	−0.13	(−0.38,	0.12)	28.3	(5.9)	28.6	(6.3)	**−0.28**	**(−0.45,**	**−0.11)**	27.1	(5.2)	27.4	(5.5)	**−0.32**	**(−0.46,**	**−0.18)**

Boldface indicates mean differences with a *p* value < 0.05.

*SD* standard deviation, *MD* mean difference, *kg* kilograms, *cm* centimeters, *kg/m*^*2*^ kilograms/meters^2^.

By educational attainment, underreporting of weight was similarly higher among White and Latina participants who completed some college or ≥college compared to White and Latina participants who completed ≤high school (Table 3). Underreporting of weight was highest among Black participants who completed ≥college who had a mean of differences between self-reports and objective measurements of −1.61 kg ([95% CI: −1.85, −1.37]). Although misreporting of height showed little variation by educational attainment among White participants, height underreporting was of higher magnitude among Black and Latina participants with higher educational attainment. However, CIs of means of differences overlapped. BMI underestimation was highest among Black participants who completed ≥college (mean of differences = −0.45 kg/m^2^ [95% CI: −0.55, −0.34]).

### Measurement error and agreement in BMI categories in relation to educational attainment

#### Weight

Compared to their within race and ethnicity counterparts with ≤high school educational attainment, participants who attained ≥college underreported their weight by 0.32 more kg (MD_White_ = −0.32 [95% CI:−0.44, −0.20]), 0.99 more kg (MD_Black_ = −0.99 [95% CI:−1.61, −0.38]), and 0.76 more kg (MD_Latina_ = −0.76 [95% CI:−1.25,−0.27]) after adjustment (p_race/ethnicity*educational attainment_ = 0.02; Table 4). Across racial and ethnic groups (p-interaction = 0.89), participants with ≥college were more likely to both underreport their weight by 7lbs/3.18 kg or more and were less likely to overreport their weight by 4 lbs/1.81 kg or more after adjustment (Supplementary Table 3). Similarly, there were no differences by race and ethnicity in relationships with educational attainment when agreement in weight was assessed as percentages (p_interaction_=0.49; Table 5). Participants who attained ≥college compared to participants who attained ≤high school had higher odds of under-reporting weight by ≥5% (OR = 1.27 [95% CI:1.09-1.47]) and lower odds of over-reporting weight by 5% or more (OR = 0.59 [95% CI:0.47-0.75]).

**Table 4:** Associations between educational attainment and measurement error of weight, height, and derived body mass index (BMI), overall and within racial and ethnic groups, Sister Study (2003–2009), N = 18,368.

All participants									
Predictors	SR–OM Weight (kg)^a^			SR–OM Height (cm)			SR–OM BMI (kg/m^2^)		
	Estimates	CI	*p*	Estimates	CI	*p*	Estimates	CI	*p*
Some college vs. ≤High school	−0.19	−0.31 to −0.07	**0.002**	−0.02	−0.11 to 0.07	0.685	−0.07	−0.13 to −0.02	**0.008**
≥College vs. ≤High school	−0.39	−0.51 to −0.28	**<0.001**	0.07	−0.01 to 0.16	0.101	−0.18	−0.23 to −0.13	**<0.001**
Observations	18,368			18,368			18,368		
**White participants**									
	**SR–OM Weight (kg)**			**SR–OM Height (cm)**			**SR–OM BMI (kg/m**^**2**^**)**		
**Predictors**	**Estimates**	**CI**	***p***	**Estimates**	**CI**	***p***	**Estimates**	**CI**	***p***
Some college vs. ≤High school	−0.14	−0.26 to −0.01	**0.030**	0.02	−0.08 to 0.11	0.745	−0.07	−0.12 to −0.01	**0.019**
≥College vs. ≤High school	−0.32	−0.44 to −0.20	**<0.001**	0.07	−0.02 to 0.16	0.112	−0.15	−0.21 to −0.10	**<0.001**
Observations	15,502			15,502			15,502		
**Black participants**									
	**SR–OM Weight (kg)**			**SR–OM Height (cm)**			**SR–OM BMI (kg/m**^**2**^**)**		
**Predictors**	**Estimates**	**CI**	***p***	**Estimates**	**CI**	***p***	**Estimates**	**CI**	***p***
Some college vs. ≤High school	−0.57	−1.20 to 0.06	0.078	−0.15	−0.60 to 0.30	0.523	−0.15	−0.43 to 0.13	0.284
≥College vs. ≤High school	−0.99	−1.61 to −0.38	**0.002**	0.07	−0.37 to 0.50	0.768	−0.38	−0.65 to −0.11	**0.005**
Observations	1857			1857			1857		
**Latina participants**									
	**SR–OM Weight (kg)**			**SR–OM Height (cm)**			**SR–OM BMI (kg/m**^**2**^**)**		
**Predictors**	**Estimates**	**CI**	***p***	**Estimates**	**CI**	***p***	**Estimates**	**CI**	***p***
Some college vs. ≤High school	−0.71	−1.21 to −0.21	**0.005**	−0.36	−0.81 to 0.10	0.124	−0.16	−0.41 to 0.09	0.205
≥College vs. ≤High school	−0.76	−1.25 to −0.27	**0.002**	0.11	−0.34 to 0.56	0.633	−0.33	−0.57 to −0.08	**0.008**
Observations	1009			1009			1009		

Boldface values indicate statistical significance at a two-sided *p*-value of 0.05. Models are adjusted for age (years), marital status (married/living as married, single/never married, divorced/separated/widowed), objectively-measured height (cm), BMI category (underweight, recommended, overweight, Class I obesity, Class II obesity, Class III obesity), weight fluctuations over the life course (never, 1–2 times, ≥3 times), perceived health status (fair/poor: yes vs. no), anti-depressant use (yes, no), and the time between in-home exam and CATI (exam and self-report within 30 days, exam >30 days after self-report). Models for all participants are additionally adjusted for race and ethnicity.

*SR* self-reported, *OM* objectively measured, *kg* kilograms, *cm* centimeters, *kg/m*^*2*^ kilograms/meters^2^, *CI* confidence interval.

^a^Wald *p*-interaction (educational attainment*race/ethnicity) = 0.02 for measurement error of weight.

**Table 5:** Associations between educational attainment and under- as well as over-reporting of weight by 5%, overall and across racial and ethnic groups, Sister Study (2003–2009), N = 18,368.

	n	Under-report by ≥5% vs. Report within 5%			Over-report by ≥5% vs. Report within 5%		
		Odds ratio (95% Confidence interval)					
Overall	18,638						
≤High School	2969	1.00	reference		1.00	reference	
Some College	6365	1.12	(0.96,	1.30)	0.78	(0.61,	1.00)
≥College	9034	**1.27**	**(1.09,**	**1.47)**	**0.59**	**(0.47,**	**0.75)**
White	15,502						
≤High School	2591	1.00	reference		1.00	reference	
Some College	5323	1.09	(0.93,	1.29)	0.81	(0.61,	1.06)
≥College	7588	**1.26**	**(1.07,**	**1.48)**	**0.66**	**(0.51,**	**0.87)**
Black	1857						
≤High School	159	1.00	reference		1.00	reference	
Some College	679	1.03	(0.60,	1.78)	0.52	(0.25,	1.11)
≥College	1019	1.13	(0.67,	1.91)	**0.27**	**(0.12,**	**0.58)**
Hispanic/Latina	1009						
≤High School	219	1.00	reference		1.00	reference	
Some College	363	1.64	(0.86,	3.12)	0.83	(0.38,	1.84)
≥College	427	1.50	(0.80,	2.84)	0.43	(0.18,	1.01)

Models are adjusted for age (years), marital status (married/living as married, single/never married, divorced/separated/widowed), objectively-measured height (cm), BMI category (underweight, recommended, overweight, Class I obesity, Class II obesity, Class III obesity), weight fluctuations over the life course (never, 1–2 times, ≥3 times), perceived health status (fair/poor: yes vs. no), anti-depressant use (yes, no), and the time between in-home exam and CATI (exam and self-report within 30 days, exam >30 days after self-report). Models for all participants are additionally adjusted for race/ethnicity. Boldface values indicate significance at a two-sided *p*-value of 0.05. Likelihood ratio test two-sided *p*-value comparing models with and without race/ethnicity*educational attainment interaction term = 0.49.

#### Height

Compared to participants with ≤high school educational attainment, misreporting of height was similar to misreporting among participants who attained either some college (MD = -0.02 [95% CI:-0.11,0.07]) or ≥college (MD = 0.07 [95% CI:-0.01,0.16]; Table 4). This misreporting did not differ by race and ethnicity (p_interaction_>0.05). Compared to participants who attained ≤high school, White, Black, and Latina participants with ≥college were similarly less likely to either underreport or overreport their height by ≥1 inch/2.54 cm (p_interaction_ = 0.49; Table 6). Overall, participants who attained ≥college were 24% less likely to underreport their height by ≥1 inch/2.54 cm (OR = 0.76 [95% CI:0.67,0.87]) and were 15% less likely to overreport their height by ≥1 inch/2.54 cm (OR = 0.85 [95% CI:0.73,0.98]).

**Table 6:** Associations between educational attainment and under- as well as over-reporting of height, overall and across racial and ethnic groups, Sister Study (2003–2009), N = 18,368.

	n	Under-report by ≤1 inch (2.54 cm) vs. report within 1 inch (2.54 cm)			Over-report by ≥1 inch (2.54 cm) vs. report within 1 inch (2.54 cm)		
		Odds ratio (95% Confidence interval)					
Overall	18,638						
≤High School	2969	1.00	reference		1.00	reference	
Some College	6365	0.91	(0.80,	1.04)	0.89	(0.77,	1.04)
≥College	9034	**0.76**	**(0.67,**	**0.87)**	**0.85**	**(0.73,**	**0.98)**
White	15,502						
≤High School	2591	1.00	reference		1.00	reference	
Some College	5323	**0.86**	**(0.75,**	**1.00)**	0.93	(0.79,	1.10)
≥College	7588	**0.75**	**(0.66,**	**0.86)**	0.88	(0.75,	1.03)
Black	1857						
≤High School	159	1.00	reference		1.00	reference	
Some College	679	1.39	(0.83,	2.33)	0.72	(0.41,	1.28)
≥College	1019	0.96	(0.57,	1.60)	0.65	(0.37,	1.13)
Hispanic/Latina	1009						
≤High School	219	1.00	reference		1.00	reference	
Some College	363	1.10	(0.65,	1.84)	0.69	(0.41,	1.16)
≥College	427	0.74	(0.44,	1.25)	0.79	(0.48,	1.31)

Models are adjusted for age (years), marital status (married/living as married, single/never married, divorced/separated/widowed), objectively-measured height (cm), BMI category (underweight, recommended, overweight, Class I obesity, Class II obesity, Class III obesity), weight fluctuations over the life course (never, 1–2 times, ≥3 times), perceived health status (fair/poor: yes vs. no), anti-depressant use (yes, no), and the time between in-home exam and CATI (exam and self-report within 30 days, exam >30 days after self-report). Models for all participants are additionally adjusted for race and ethnicity. Boldface values indicate significance at a two-sided *p*-value of 0.05. Likelihood ratio test two-sided *p*-value comparing models with and without race/ethnicity*educational attainment interaction term = 0.49.

#### BMI

Participants with higher educational attainment had higher underestimation when continuous BMI was derived using self-reports vs. objective measurements; there was no variation by race and ethnicity (p_interaction_>0.05; Table 4). Compared to participants who attained ≤high school, derived BMI was underestimated by 0.07 more kg/m^2^ (MD = -0.07 [95% CI:-0.13,-0.02]) among participants with some college and by 0.18 more kg/m^2^ (MD = -0.18 [-0.23, -0.13]) among participants with ≥college after adjustment. Overall, obesity prevalence was 29% when BMI was derived from self-reported height and weight and was 31% when derived from objective measurement (agreement=83%, weighted *k* = 0.93, 88% sensitivity, 97% specificity; Supplementary Tables 4 and 5). Within groups, sensitivity ranged from 84% (Latina with ≥college) to 93% (Black and Latina women with ≤high school), and specificity ranged from 90% (Latina women with ≤high school) to 99% (White women with ≥college).

Agreement between BMI categories did not vary by educational attainment categories among the overall population or White women; however, agreement between BMI categories varied by educational attainment among Black and Latina women (Supplementary Tables 6–9). Although CIs overlapped between weighted kappas, BMI category agreement was lowest among Black participants who attained ≤high school (74% agreement, weighted *k* = 0.90) compared to BMI category agreement observed among Black participants with some college (77% agreement, weighted *k* = 0.92) and ≥college (80% agreement, weighted k = 0.92). Among Black women, obesity prevalence based on derived BMI from self-reported and objective measurements, respectively, was 58% and 60% among participants who completed ≤high school, 53% and 57% among participants who completed some college, and 45% and 48% among participants who completed ≥college. Although kappa CIs also overlapped among Latina participants, agreement between categorical BMI measures was weaker in the lower compared to higher educational attainment categories: ≤high school (78% agreement, weighted *k* = 0.89), some college (77% agreement, weighted *k* = 0.92), and ≥college (84% agreement, weighted k = 0.93).

### Secondary analysis

The results among 46,618 participants who completed self-reports either prior to or after objective measurements were generally consistent with the primary analysis (Supplementary Tables 10–16). Generally, means of differences and limits of agreement estimated in Bland-Altman analysis were similar but slightly smaller than those observed in the main analysis. Mean differences between self-reported and objectively measured weight and BMI were consistent with the main results: the highest underreporting of weight was among Black participants who attained ≥college. Additionally, across educational attainment categories, agreement in continuous measures of weight remained good, agreement in continuous height was mostly fair, and agreement in continuous BMI remained good among White participants and mostly fair among Black and Latina participants. Lastly, agreement in categorical BMI measurements remained high, nearly perfect, and similar across race and ethnicity and educational attainment categories.

## Discussion

In the ongoing Sister Study cohort, White, Black, and Latina women generally underreported their weight and height, which resulted in an underestimation of derived BMI. Further, as expected, women with the highest vs. lowest levels of educational attainment were more likely to underreport weight and consistent with our hypothesis, the magnitude of these differences, when measured continuously, was largest among Black women. Also consistent with our hypothesis, there were no differences in height measurement error by race and ethnicity, and women with the highest vs. lowest educational attainment were less likely to misreport height by 2.54 cm or more. Across educational attainment categories within racial and ethnic groups, there was fair to good agreement between self-reported and objective continuous measures of weight, height, and BMI. Obesity prevalence was underestimated by approximately 2% across races and ethnicities. Nonetheless, agreement between self-reported and examiner/objectively-measured BMI categories was almost perfect across racial and ethnic groups and educational attainment categories within racial and ethnic groups [41]. Therefore, despite reporting error in weight, height, and derived BMI, categorical measures of self-reported BMI are likely a good metric of obesity status among Sister Study participants across racial and ethnic groups and educational attainment categories.

Women with higher versus lower educational attainment (i.e., ≥college vs. ≤high school) were more likely to underreport their weight—with the largest differences in continuous measurements among Black and Latina women—but were less likely to misreport their height by 2.54 cm or more. Prior studies have shown differences in misreporting by socioeconomic status [15, 17, 18, 22]; however, directions of associations have been mixed. For instance, while some prior studies have found lower mis- or under-reporting of weight among women with higher educational attainment [18, 22], another study reported that women with higher socioeconomic status (SES) (i.e., ≥some college) were more likely to underreport their weight compared to women with lower SES (i.e., no high school diploma and annual income <$20,000) [17]. However, the agreement between self-reported and objectively measured weight did not vary by SES in that study, similar to our findings for BMI categories. Likewise, a more recent study reported that SES (educational attainment and income) was not associated with misreporting of weight or height in a sample from the United Kingdom [43]. Yet, misreporting of weight and height has been found to lead to higher underestimation of BMI among women with higher educational attainment in studies using National Health and Nutrition Examination Survey data [17, 19]. Varying results across studies are likely related to differing populations as well as the measures and categorizations of SES factors.

Associations between race and ethnicity with accuracy of self-reported weight, height, and derived BMI have also been mixed [13, 17–19, 22–25, 44]. For instance, while some previous studies have found that Black and Latina women are less likely to have underreported derived BMI than White individuals [17, 19], prior studies also corroborate our findings that Black participants were more likely to underreport weight compared to White women [22, 23]. Other studies do not suggest significant differences between Black and White women in underreporting of weight [13, 18, 25, 44]. Our study considered the intersection of race and ethnicity with educational attainment in relation to the accuracy of self-reported weight, height, and derived BMI. We found similar associations between educational attainment and misreporting for all measurements across racial and ethnic groups except for larger errors in self-reporting of continuous weight among Black and Latina compared to White participants. Further study is warranted to determine relationships in other samples.

Clinical and psychosocial factors may explain our observation of underreporting of weight that increased with educational attainment and its variation by race and ethnicity. Black participants had the highest differences between self-reported and measured weight, and the magnitude of underreporting of weight by educational attainment was larger among Black compared to White participants. Prior studies suggest that misreporting of weight is higher among people with obesity [7, 9, 15, 18, 22, 23]. Obesity prevalence was higher among Black and Latina participants. Coupled with the higher prevalence of obesity across educational attainment categories among Black participants, the higher likelihood of underperceiving weight among Black vs. White women due to racial and ethnic differences in educational attainment, body image norms, and perceived social desirability [23, 45–47] likely contributes to the higher underreporting among Black women in our sample. For instance, Black women with higher educational attainment in higher status occupations may encounter more pressure to conform to Western norms than Black women with lower educational attainment, resulting in higher underreporting of weight.

Similar to our study, previous studies have found that self-reported weight and height among women often leads to underestimates of BMI [6, 8, 9, 14, 22, 48]; however, this underestimation is negligible for epidemiological purposes, as agreement between self-reported and objectively measured weight and height are often high, resulting in accurate estimates of BMI categories [8, 16, 22, 25, 49]. Similarly, our study supports the accuracy of the BMI category derived from self-reported height and weight in the Sister Study, as demonstrated by the near-perfect agreement with objectively measured BMI category across demographic groups.

Our study has limitations. We assessed measurement error cross-sectionally and, therefore, cannot determine the direction of the relationships between educational attainment and perceptions of anthropometrics. The time between the CATI and exam differed by race and ethnicity, which may have affected results; however, most women completed the CATI and reported anthropometrics within 30 days of objective measurements. Additionally, knowledge that a home visit, which included biometrics assessment, would follow the CATI may have impacted self-reports and related bias. Residual confounding is possible due to the observational nature of the study. Our study was limited to self-identified White, Black, and Latina women; however, further study is warranted among populations of women with greater racial and ethnic diversity.

Further, the Sister Study is a unique cohort of women with sisters who had breast cancer that is primarily White with higher socioeconomic status, which limits generalizability. Lastly, we note that the use of BMI, although of utility for epidemiologic purposes, is inherently biased [50] since it was initially developed as an index to determine “normal” relative weight among European males [51]. The simplicity of using BMI as a measure of obesity across racial and ethnic groups does not reflect fat distribution, which is a more sensitive measure of obesity-related adverse health risk that has been shown to vary by race and ethnicity among women with similar BMIs [52]. Nonetheless, the study is strengthened by using a large, heterogenous cohort of women with adequate power to investigate measurement error by educational attainment.

Our study supports previous literature that demonstrates self-reported height and weight as practical measures to determine BMI, especially BMI category, which has important public health implications in estimating burdens of obesity and longitudinal associations with obesity in an ongoing national cohort. Although there were measurement error differences between groups, BMI category based on self-reports was largely unbiased. Specifically, Sister Study participants with lower compared to higher educational attainment, a subgroup burdened by higher rates of obesity [26], were less likely to underreport their weight. Conversely, Black women, a subgroup also disproportionately burdened by obesity [27], underreport weight to a greater degree than their White counterparts. Despite these differences, agreement between BMI categories derived from self-report and measured weight and height was near perfect across racial and ethnic groups, which supports the use of BMI categories based on self-reports for epidemiological and public health purposes in this large cohort of over 50,000 women for which repeated measurement is costly and complex.

## Conclusion

BMI category based on self-reported weight and height appears to be an accurate, generally unbiased measure among women enrolled in the Sister Study across educational attainment categories, overall and within racial and ethnic groups. Although there was evidence of measurement error and some differences in measurement error by educational attainment both overall and within racial and ethnic groups, measurement error did not preclude the reliability of self-reported measures of BMI category in this cohort. Essentially, self-reported BMI categorical data were highly agreeable with objective BMI categorical measures across races and ethnicities of women of various levels of socioeconomic status, supporting the utility of self-reported data in extensive cohort investigations involving overweight/obesity.

## Supplementary information

BMI Validation supplemental material

## Data Availability

Data, including anonymized data for replication, are available as described on the Sister Study website (https://sisterstudy.niehs.nih.gov/English/coll-data.htm). Data use is restricted to replication, only. New analyses must be approved after a proposal process. Code can be requested from the corresponding author.
