# Supplementary material for: Agreement between self-reported and measured weight, height, and derived BMI by educational attainment across racial and ethnic groups of US women
Source: Int J Obes (Lond). 2025 Apr 14;49(8):1543–53. doi: 10.1038/s41366-025-01784-8 (PMC12353154; doi:10.1038/s41366-025-01784-8)
Supplement: Supplementary file 1 — BMI Validation supplemental material [file 41366_2025_1784_MOESM1_ESM.pdf]

Description of Supplemental Material

Supplemental Figure 1. Flow Chart Diagram for the Analytic Sample, Sister Study

Supplemental Figure 2. Bland−Altman Plot: Weight by Educational Attainment among (a) White, (b) Black, and (c) Latina Women

Supplemental Figure 3. Bland−Altman Plot: Height by Educational Attainment among (a) White, (b) Black, and (c) Latina Women

Supplemental Figure 4. Bland−Altman Plot: BMI by Educational Attainment among (a) White, (b) Black, and (c) Latina Women

Supplemental Table 1. Comparison of characteristics at enrollment among excluded and included participants, Sister Study (2003-2009), N=50,884

Supplemental Table 2. R Packages used in Analysis

Supplemental Table 3. Associations between educational attainment and under- and over-reporting of weight in pounds/kilograms, overall and across racial and ethnic groups, Sister Study (2003-2009), N=18,638

Supplemental Table 4. Proportions, percentage agreements, kappas, and weighted kappas for categorical body mass index (BMI) among the overall population and by race and ethnicity, Sister Study (2003-2009), N=18,368

Supplemental Table 5. Sensitivity and specificity of obesity status (BMI ≥30 kg/m2), overall, by educational attainment, and by educational attainment within racial and ethnic groups, Sister Study, (2003-2009), N=18,368

Supplemental Table 6. Proportions, percentage agreements, kappas, and weighted kappas for categorical body mass index (BMI) among the overall population and by educational attainment, Sister Study (2003-2009), N=18,368

Supplemental Table 7. Proportions, percentage agreement, kappa, and weighted kappa for categorical body mass index (BMI) among the overall population and by educational attainment among non-Hispanic White participants, Sister Study (2003-2009), N=15,502

Supplemental Table 8. Proportions, percentage agreement, kappa, and weighted kappa for categorical body mass index (BMI) among the overall population and by educational attainment among Black/African American participants, Sister Study (2003-2009), N=1,857

Supplemental Table 9. Proportions, percentage agreement, kappa, and weighted kappa for categorical body mass index (BMI) among the overall population and by educational attainment among Latina participants, Sister Study (2003-2009), N=1,009

Supplemental Table 10. Bland-Altman plot statistics: Bias (mean of differences between self-report and objective measures) and limits of agreement by educational attainment within racial and ethnic groups among participants who completed self-reports either prior to or after objective measurements, Sister Study (2003-2009), N=46,618

Supplemental Table 11. Mean differences between self-reported and objectively/examiner measured weight, height, and body mass index, overall and by educational attainment among participants who completed self-reports either prior to or after objective measurements within the overall population and within racial and ethnic groups, Sister Study (2003-2009), N=46,618

Supplemental Table 12. Proportions, percentage agreement, kappa, and weighted kappa for categorical body mass index (BMI) among participants who completed self-reports either prior to or after objective measurements, overall and by race and ethnicity, Sister Study (2003-2009), N=46,618

Supplemental Table 13. Proportions, percentage agreement, kappa, and weighted kappa for categorical body mass index (BMI) among participants who completed self-reports either prior to or after objective measurements, overall and by educational attainment, Sister Study (2003-2009), N=46,618

Supplemental Table 14. Proportions, percentage agreement, kappa, and weighted kappa for categorical body mass index (BMI) among non-Hispanic White participants who completed self-reports either prior to or after objective measurements, overall and by educational attainment, Sister Study (2003-2009), N=40,145

Supplemental Table 15. Proportions, percentage agreement, kappa, and weighted kappa for categorical body mass index (BMI) among non-Hispanic Black/African American participants who completed self-reports either prior to or after objective measurements, overall and by educational attainment, Sister Study (2003-2009), N=4,156

Supplemental Table 16. Proportions, percentage agreement, kappa, and weighted kappa for categorical body mass index (BMI) among Hispanic/Latina participants who completed self-reports either prior to or after objective measurements, overall and by educational attainment, Sister Study (2003-2009), N=2,317

Supplemental Figure 1. Flow Chart Diagram for the Analytic Sample, Sister Study

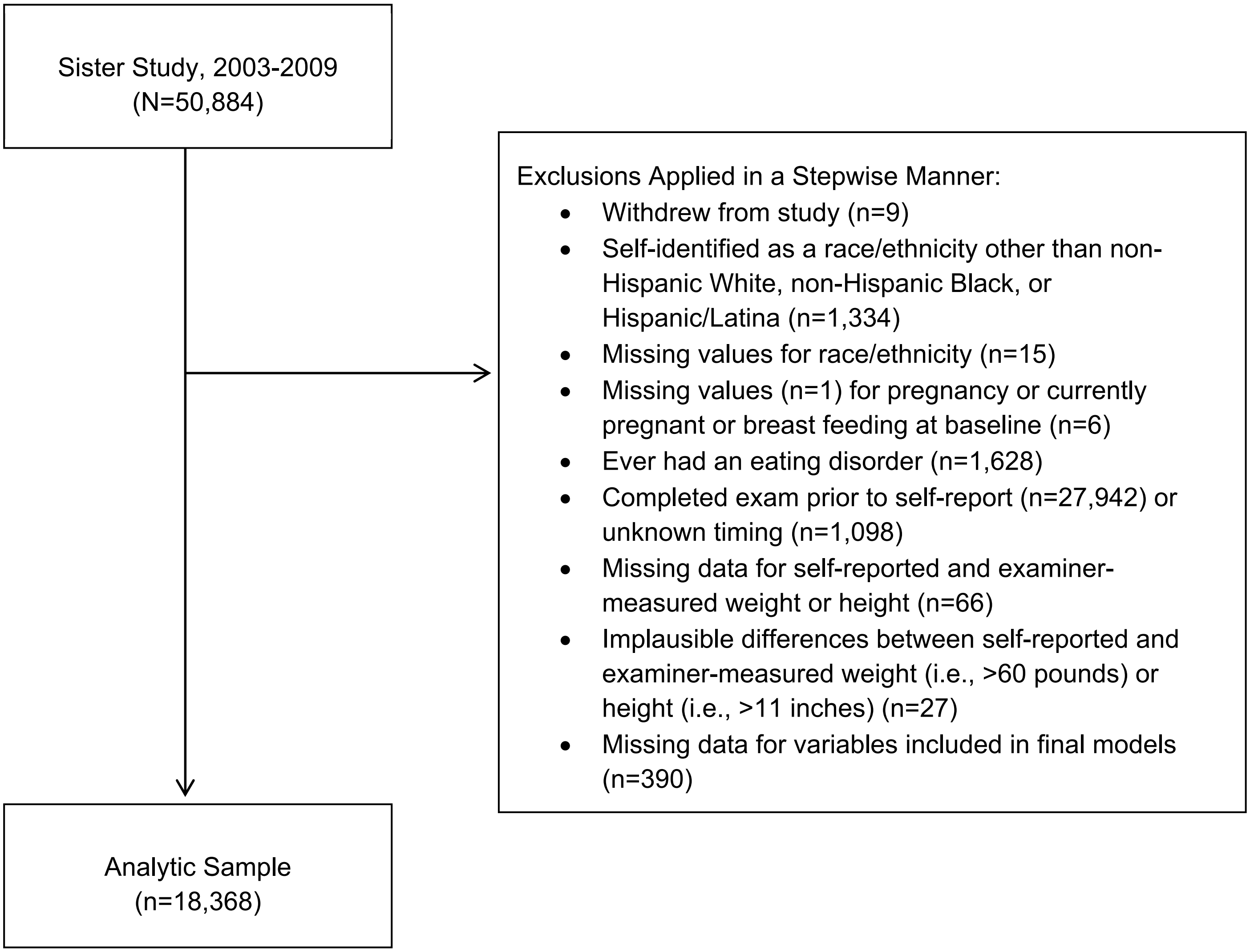

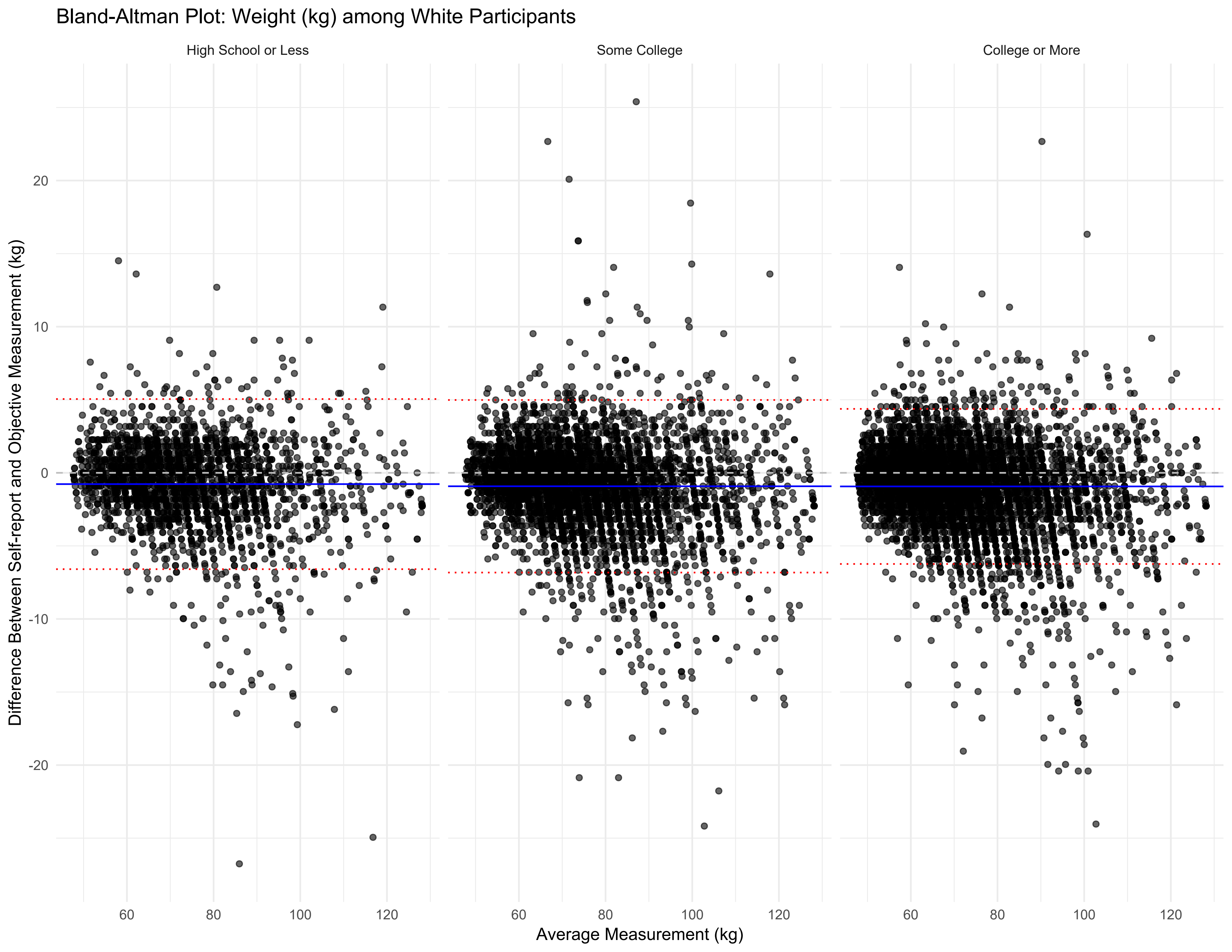
Bland-Altman Plot: Weight (kg) among White Participants
High School or Less
Some College
College or More
Difference Between Self-report and Objective Measurement (kg)
20
10
0
-10
-20
60
80
100
120
Average Measurement (kg)

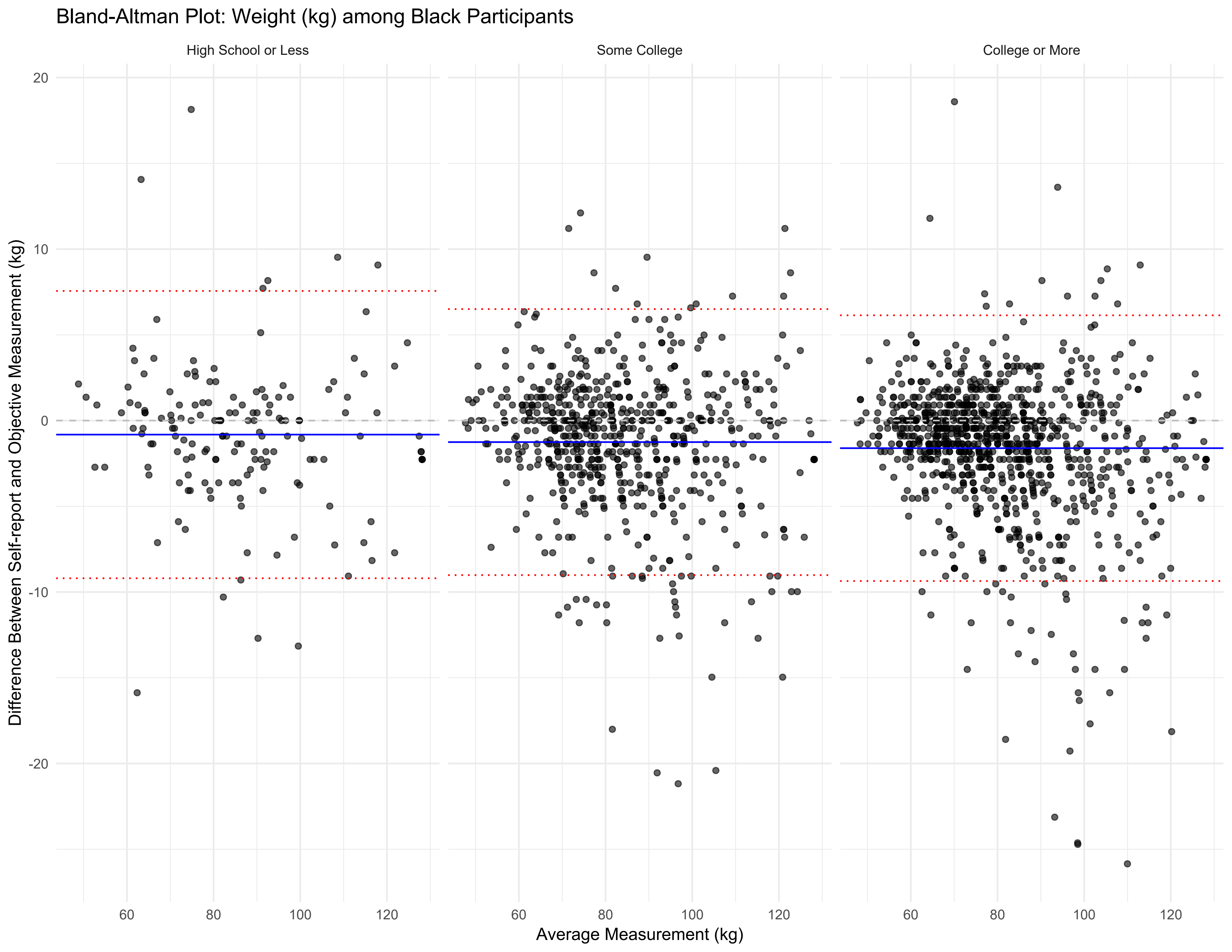
Bland-Altman Plot: Weight (kg) among Black Participants
High School or Less
Some College
College or More
Difference Between Self-report and Objective Measurement (kg)
20
10
0
-10
-20
60
80
100
120
Average Measurement (kg)

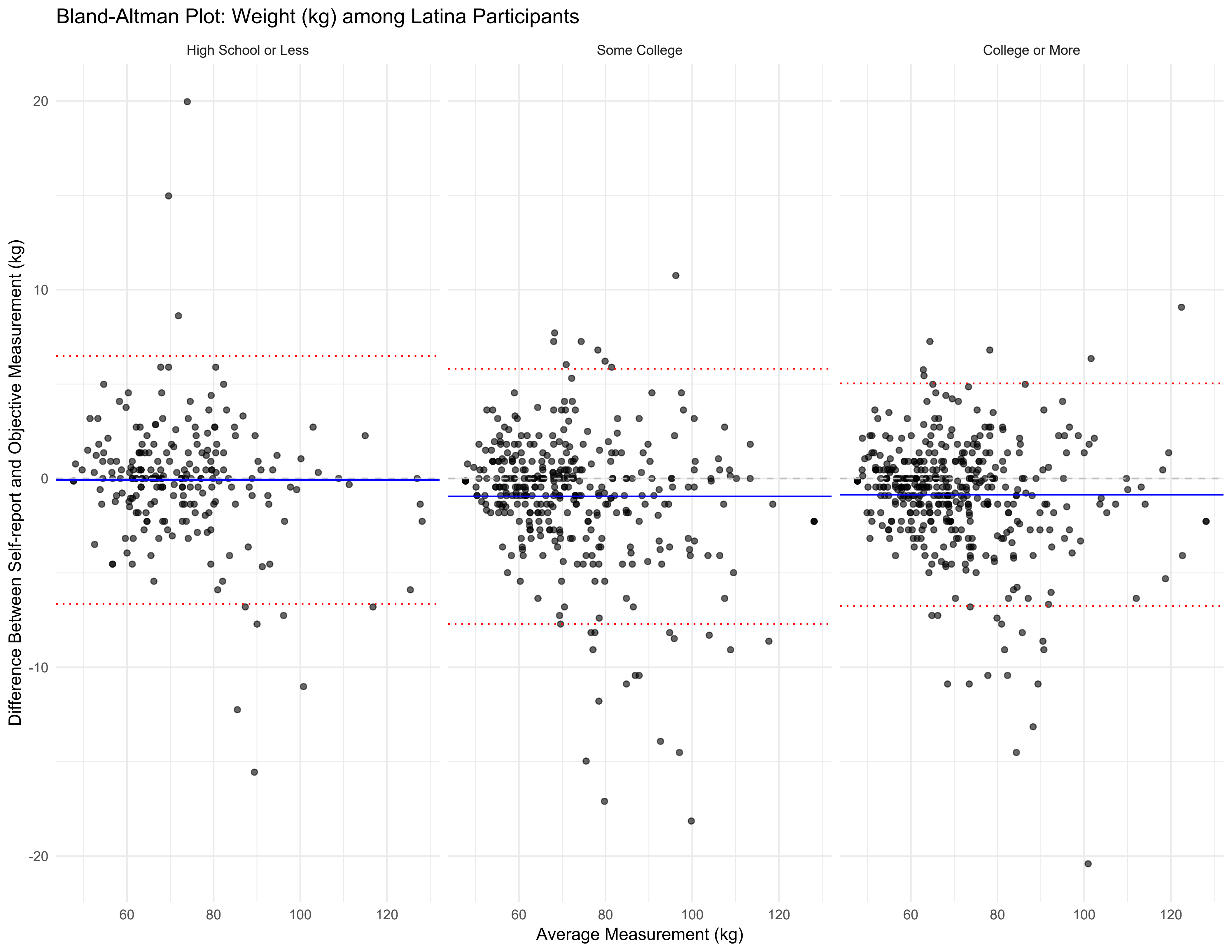
Bland-Altman Plot: Weight (kg) among Latina Participants
High School or Less
Some College
College or More
Difference Between Self-report and Objective Measurement (kg)
20
10
0
-10
-20
60
80
100
120
Average Measurement (kg)

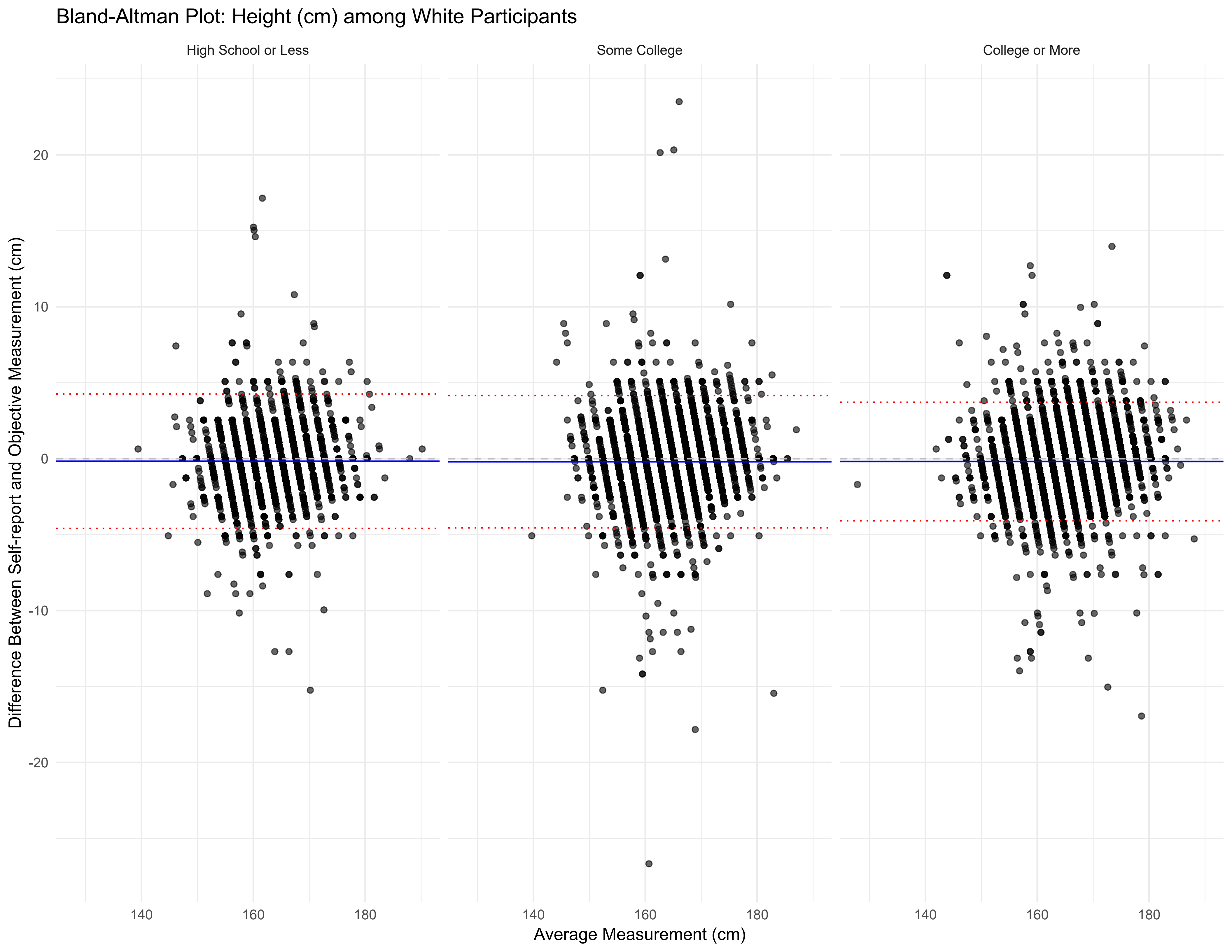

Bland-Altman Plot: Height (cm) among White Participants
High School or Less
Some College
College or More
Difference Between Self-report and Objective Measurement (cm)
20
10
0
-10
-20
140
160
180
Average Measurement (cm)

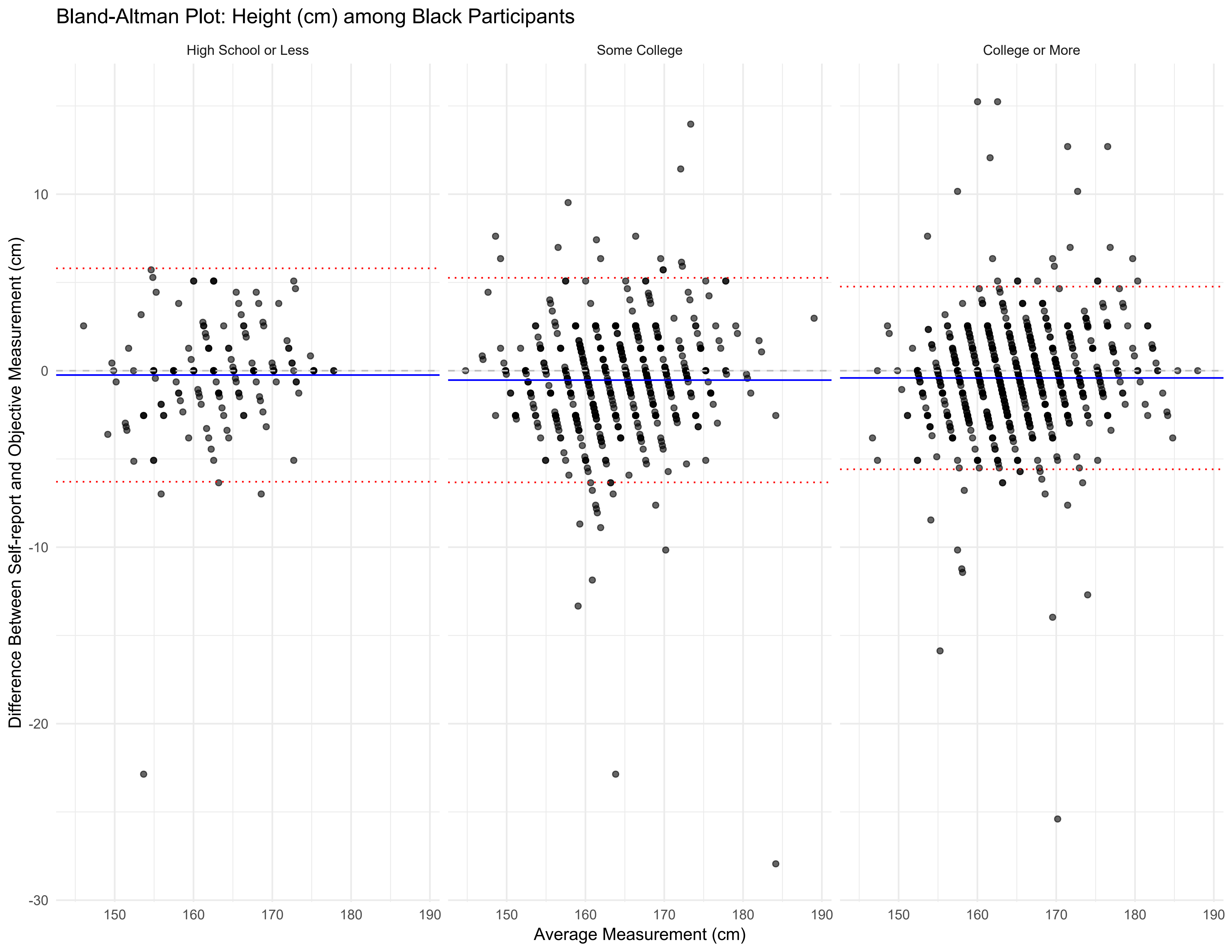

Bland-Altman Plot: Height (cm) among Black Participants
High School or Less
Some College
College or More
Difference Between Self-report and Objective Measurement (cm)
Average Measurement (cm)
10
0
-10
-20
-30
150
160
170
180
190

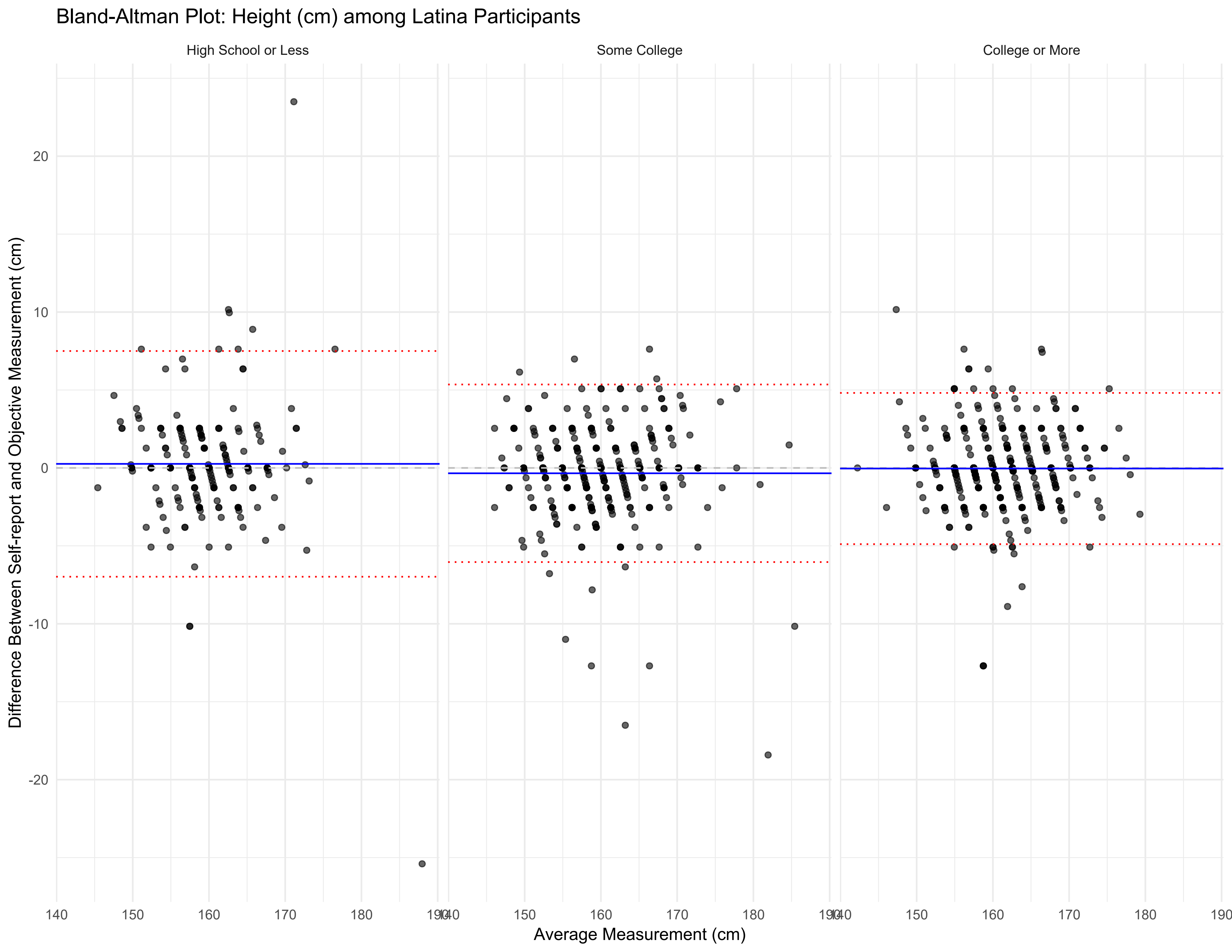

Bland-Altman Plot: Height (cm) among Latina Participants
High School or Less
Some College
College or More
Difference Between Self-report and Objective Measurement (cm)
20
10
0
-10
-20
140
150
160
170
180
190
Average Measurement (cm)

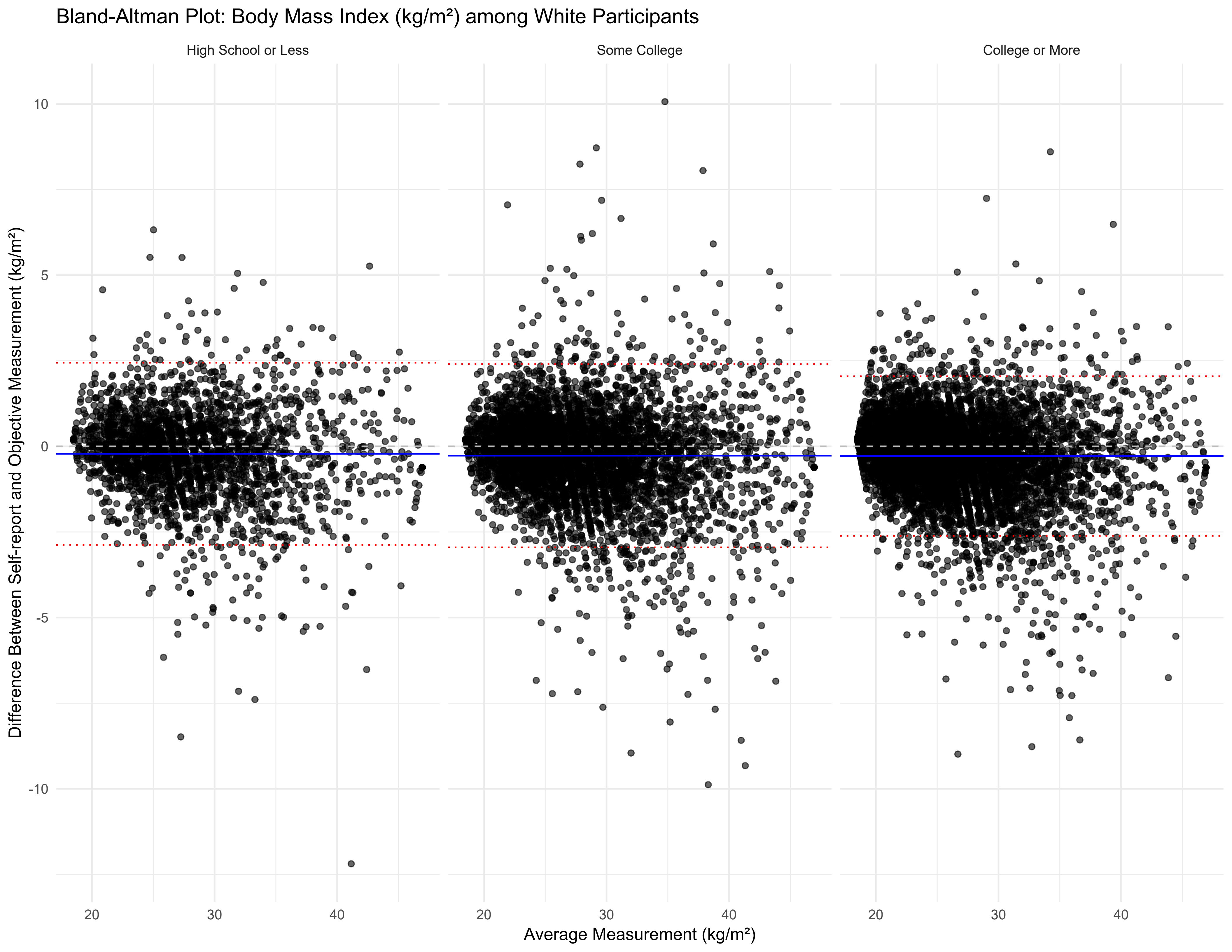
Bland-Altman Plot: Body Mass Index (kg/m²) among White Participants
High School or Less
Some College
College or More
Difference Between Self-report and Objective Measurement (kg/m²)
10
5
0
-5
-10
20
30
40
Average Measurement (kg/m²)

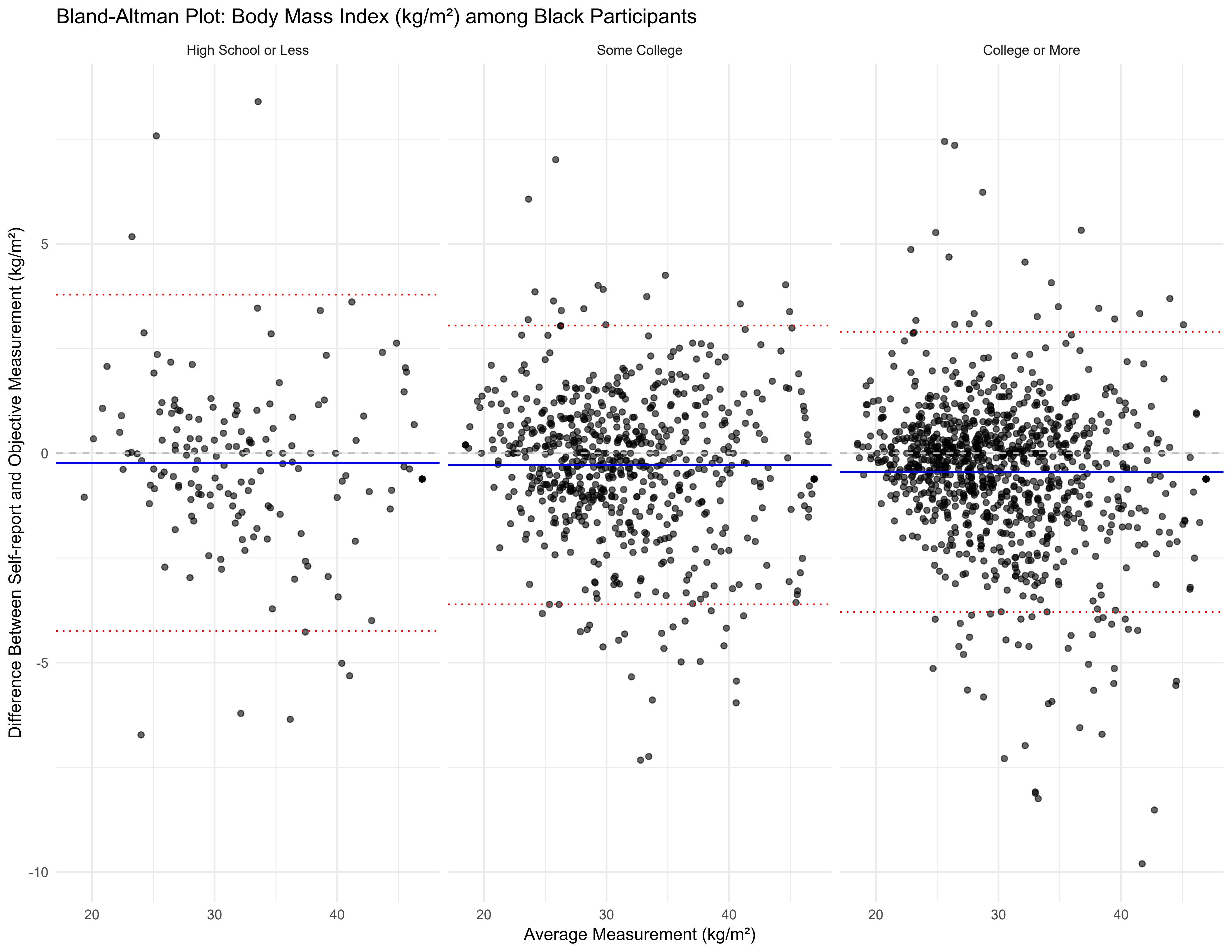

Bland-Altman Plot: Body Mass Index (kg/m²) among Black Participants
High School or Less
Some College
College or More
Difference Between Self-report and Objective Measurement (kg/m²)
5
0
-5
-10
20
30
40
Average Measurement (kg/m²)

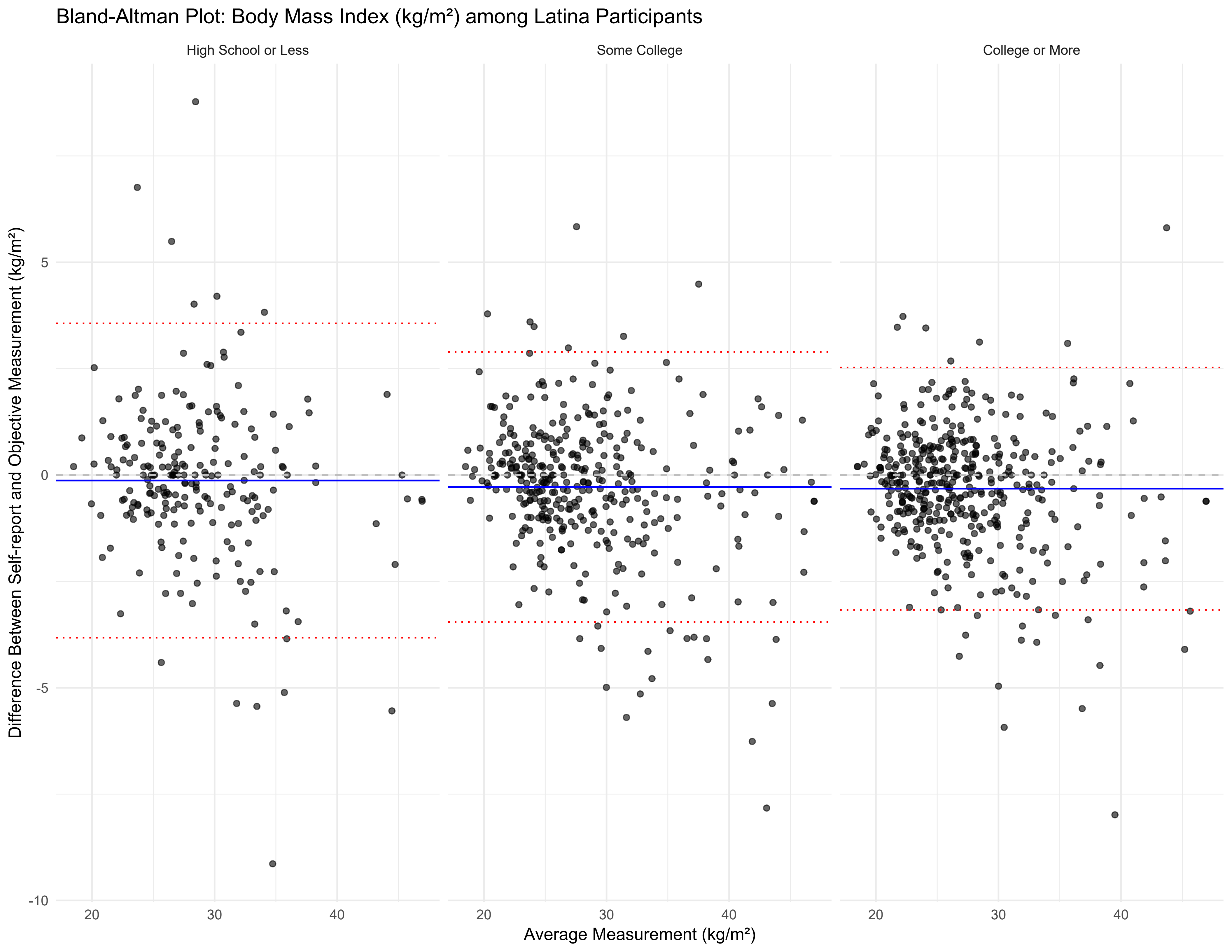

Bland-Altman Plot: Body Mass Index (kg/m²) among Latina Participants
High School or Less
Some College
College or More
Difference Between Self-report and Objective Measurement (kg/m²)
5
0
-5
-10
20
30
40
Average Measurement (kg/m²)

Supplemental Table 1. Comparison of characteristics at enrollment among excluded and included participants, Sister Study (2003-2009), N=50,884

	**Total**	**Excluded**	**Included**	**P-value [a]**
N or n	50,884	32,516	18,368	
**Sociodemographic Characteristics**	**%, mean (SD), or n missing**			
Age, years	55.2 (9.0)	54.9 (9.0)	55.7 (9.0)	<0.001
Age categories				<0.001
35-64 years	83	84	81	
65+ years	17	16	19	
Race/ethnicity				<0.001
Non-Hispanic White	84	83	84	
Non-Hispanic Black	9	8	10	
Hispanic/Latina	5	5	5	
Other Race and Ethnicity [b]	3	4	0	
n Missing	15	15	0	
Educational attainment				<0.001
≤High School	15	15	16	
Some College	34	33	35	
Bachelor's Degree/≥College	51	52	49	
n Missing	21	21	0	
Annual household income				0.007
<$20,000	5	5	5	
$20,000 - $50,000	21	21	22	
$50,000 - $99,000	41	41	41	
≥$100,000	33	34	33	
n Missing	9	9	0	
Marital status				0.1
Married/living as married	75	75	74	
Single/never married	5	5	6	
Divorced/separated/widowed	20	20	20	
n Missing	23	23	0	
**Clinical Characteristics**				

Self-reported weight, kilograms (kg)	74.0 (16.9)	73.8 (17.0)	74.3 (16.8)	0.002
n Missing	155	155	0	
Objectively measured weight, kilograms (kg)	74.9 (17.5)	74.7 (17.6)	75.2 (17.3)	<0.001
n Missing	25	25	0	
Self-reported vs. measured weight				<0.001
Under-report by ≥7 pounds (3.18 kg)	15	13	17	
Under-report by 4-6 pounds (1.81-<3.18 kg)	15	14	15	
Report within 4 pounds (1.81 kg)	60	63	54	
Over-report by ≥4 pounds (1.81 kg)	11	10	14	
n Missing	155	155	0	
Self-reported vs. measured weight				<0.001
Under-report by ≥5%	10	9	13	
Report within 5%	87	88	84	
Over-report by ≥5%	3	3	4	
n Missing	155	155	0	
Weight fluctuations (≥20 lbs/9.07 kg) over the life course				0.006
Never	53	54	53	
1-2 times	26	26	25	
≥3 times	21	21	22	
n Missing	355	355	0	
Self-reported height, centimeters (cm)	164.0 (6.6)	164.0 (6.5)	164.0 (6.6)	0.5
n Missing	42	42	0	
Objectively measured height, inches (cm)	164.2 (6.4)	164.2 (6.4)	164.2 (6.4)	0.7
n Missing	11	11	0	
Self-reported vs. measured height				<0.001
Underreporting by ≥ 1 in. (≥ 2.5 cm)	13	11	15	
Report within 1 in.	78	81	74	
Over-report by ≥ 1 in. (≥ 2.5 cm)	9	8	11	
n Missing	42	42	0	
Self-reported BMI, kg/m^2	27.6 (6.1)	27.5 (6.1)	27.7 (6.0)	0.003
n Missing	185	185	0	
Objectively measured BMI, kg/m^2	27.8 (6.3)	27.7 (6.3)	27.9 (6.2)	<0.001
n Missing	26	26	0	

BMI category based on self-report				<0.001
Underweight (BMI <18.5 kg/m^2)	1	1	1	
Recommended (18.5 kg/m^2 ≤ BMI ≤ 24.9 kg/m^2)	39	39	38	
Overweight (25 kg/m^2 ≤ BMI ≤ 29.9 kg/m^2)	32	32	32	
Obesity Class I (30 kg/m^2 ≤ BMI ≤ 34.9 kg/m^2)	17	16	18	
Obesity Class II (35 kg/m2 ≤ BMI ≤ 39.9 kg/m2)	7	7	7	
Obesity Class III (BMI ≥ 40.0 kg/m2)	4	4	4	
n Missing	185	185	0	
BMI category based on objective measurement				<0.001
Underweight (BMI <18.5 kg/m^2)	1	1	1	
Recommended (18.5 ≤ kg/m^2 ≤ BMI ≤ 24.9 kg/m^2)	37	38	36	
Overweight (25 kg/m^2 ≤ BMI ≤ 29.9 kg/m^2)	32	32	32	
Obesity Class I (30 kg/m2 ≤ BMI ≤ 34.9 kg/m2)	17	17	18	
Obesity Class II (35 kg/m2 ≤ BMI ≤ 39.9 kg/m2)	8	8	8	
Obesity Class III (BMI ≥ 40.0 kg/m2)	5	5	5	
n Missing	26	26	0	
Fair/poor general health status (yes)	7	7	6	0.019
n Missing	27	27	0	
Anti-depressant use (yes)	22	22	21	<0.001
n Missing	597	597	0	
**Interview Characteristics**				
Time between home interview and CATI (days)				<0.001
Exam > 30 days prior to CATI	29	45	0	
Exam within 30 days of CATI	57	53	64	
Exam > 30 days after CATI	14	2	36	
n Missing	210	210	0	

Percentages may not add up to 100 due to rounding.

Abbreviations: BMI (body mass index); SD (standard deviation); CATI (computer-assisted telephone interview)

[a] Two-sided p-value for Welch two sample t-test or Pearson's Chi-squared test

[b] Races and ethnicities include American Indian/Alaska Native, Asian/Pacific Islander, multiracial, and individuals identifying as some other race and ethnicity.

Supplemental Table 2. R Packages used in Analysis

Haven	DescTools	xlsx
frequency	Hmisc	gtsummary
gt	labelled	ipw
flextable	officer	nmormt
wCorr	matrixStats	gmodels
broom	tidyverse	weights
nnet	lmtest	sjPlot
sjmisc	sjlabelled	aod
psych	irr	

Supplemental Table 3. Associations between educational attainment and under- and over-reporting of weight in pounds/kilograms, overall and across racial and ethnic groups, Sister Study (2003-2009), N=18,638

	n	Under-report by ≥7 pounds (3.18 kg) vs. Report within 4 pounds (1.81 kg)		Under-report by 4-6 pounds (1.81-<3.18 kg) vs. Report within 4 pounds (1.81 kg)		Over-report by ≥4 pounds (1.81 kg) vs. Report within 4 pounds (1.81 kg)	
		Odds Ratio (95% Confidence Interval)					
**All**	18,638						
≤High School	2,969	1.00	reference	1.00	reference	1.00	reference
Some College	6,365	1.09	(0.94, 1.25)	1.03	(0.90, 1.18)	**0.81**	**(0.70, 0.93)**
≥College	9,034	**1.21**	**(1.06, 1.39)**	1.07	(0.94, 1.21)	**0.67**	**(0.58, 0.76)**
**White**	15,502						
≤High School	2,591	1.00	reference	1.00	reference	1.00	reference
Some College	5,323	1.04	(0.89, 1.21)	1.01	(0.87, 1.16)	**0.79**	**(0.68, 0.92)**
≥College	7,588	**1.19**	**(1.03, 1.37)**	1.06	(0.92, 1.21)	**0.67**	**(0.58, 0.77)**
**Black**	1,857						
≤High School	159	1.00	reference	1.00	reference	1.00	reference
Some College	679	1.15	(0.69, 1.94)	1.04	(0.59, 1.85)	0.87	(0.51, 1.49)
≥College	1,019	1.24	(0.75, 2.05)	0.95	(0.54, 1.66)	0.64	(0.38, 1.07)
**Hispanic/Latina**	1,009						
≤High School	219	1.00	reference	1.00	reference	1.00	reference
Some College	363	**1.92**	**(1.05, 3.50)**	1.31	(0.68, 2.52)	0.92	(0.54, 1.55)
≥College	427	1.49	(0.82, 2.73)	1.48	(0.79, 2.78)	0.68	(0.40, 1.16)

Models are adjusted for age (years), marital status (married/living as married, single/never married, divorced/separated/widowed), objectively-measured height (cm), BMI category (underweight, recommended, overweight, obesity I, obesity II, obesity III), weight fluctuations over the life course (never, 1-2 times, ≥3 times), perceived health status (fair/poor: yes vs. no), anti-depressant use (yes, no), and the time between in-home exam and CATI (exam and self-report within 30 days, exam >30 days after self-report). Models for all participants are additionally adjusted for race and ethnicity.

Boldface values indicate significance at a two-sided p-value of 0.05.

Likelihood ratio test two-sided p-value comparing models with and without race/ethnicity*educational attainment interaction term= 0.89

Supplemental Table 4. Proportions, percentage agreements, kappas, and weighted kappas for categorical body mass index (BMI) among the overall population and by race and ethnicity, Sister Study (2003-2009), N=18,368

	BMI Category	Objectively/examiner measured proportion						
		Underweight	Recommended	Overweight	Obesity Class I	Obesity Class II	Obesity Class III	*Total*
		**Overall**						
Self-reported proportion	Underweight	0.6	0.2	0	0	0	0	0.8
	Recommended	0.4	33.3	3.8	0	0	0	37.5
	Overweight	0	2.3	26.5	3.5	0.1	0	32.4
	Obesity Class I	0	0	1.7	13.6	2.2	0.1	17.6
	Obesity Class II	0	0	0	0.9	5.3	0.9	7.1
	Obesity Class III	0	0	0	0	0.5	4	4.5
	Total	1.1	35.8	32.1	18.1	8.1	5	100
	Percentage Agreement	83%						
	Kappa (95% CI)	0.77	(0.76,	0.78)	Weighted Kappa (95% CI)	0.93	(0.93,	0.94)
		**White**						
	Underweight	0.7	0.2	0	0	0	0	0.9
	Recommended	0.5	35.8	3.9	0	0	0	40.2
	Overweight	0	2.2	26.3	3.4	0.1	0	32
	Obesity Class I	0	0	1.6	12.9	2.0	0.1	16.6
	Obesity Class II	0	0	0	0.7	5.0	0.8	6.5
	Obesity Class III	0	0	0	0	0.4	3.4	3.8
	Total	1.2	38.3	31.8	17.1	7.4	4.2	100
	Percentage Agreement	84%						
	Kappa (95% CI)	0.78	(0.77,	0.78)	Weighted Kappa (95% CI)	0.93	(0.93,	0.94)
		**Black**						

Underweight	0.2	0.1	0	0	0	0	0.3
Recommended	0.1	14.0	2.9	0.1	0	0	17.1
Overweight	0	2.4	26.2	4.5	0.4	0	33.5
Obesity Class I	0	0.1	1.9	20	3.8	0.1	25.9
Obesity Class II	0	0	0.1	1.9	8.9	2.4	13.3
Obesity Class III	0	0	0	0	1.2	8.8	10
Total	0.3	16.5	31.1	26.4	14.3	11.3	100
Percentage Agreement	78%						
Kappa (95% CI)	0.72	(0.69,	0.74)	Weighted Kappa (95% CI)	0.92	(0.91,	0.93)
			**Hispanic/Latina**				
Underweight	0.4	0.1	0	0	0	0	0.5
Recommended	0.3	29.2	4.8	0	0	0	34.3
Overweight	0	3.0	29.2	4.0	0	0	36.2
Obesity Class I	0	0.2	3.4	12.8	2.4	0	18.8
Obesity Class II	0	0	0	0.9	4.0	0.8	5.7
Obesity Class III	0	0	0	0	0.1	4.6	4.7
Total	0.7	32.5	37.4	17.6	6.4	5.4	100
Percentage Agreement	80%						
Kappa (95% CI)	0.72	(0.69,	0.76)	Weighted Kappa (95% CI)	0.92	(0.90,	0.93)

BMI categories are defined as follows: Underweight (BMI < 18.5 kg/m^2); Recommended (BMI 18.5 kg/m^2 to 24.9 kg/m^2); Overweight (BMI 25 kg/m^2 to 29.9 kg/m^2); Obesity Class I (BMI 30 kg/m^2 to 34.9 kg/m^2); Obesity Class II (BMI 35 kg/m^2 to 39.9 kg/m^2); and Obesity Class III (BMI ≥ 40.0 kg/m^2).

Note: Interpretations of Cohen's kappa (κ) values are no agreement (κ=0-0.20), minimal to weak agreement (0.21-0.59), moderate to strong agreement (0.60-0.90), and almost perfect agreement (≥0.90).

Supplemental Table 5. Sensitivity and specificity of obesity status (BMI ≥30 kg/m^2), overall, by educational attainment, and by educational attainment within racial and ethnic groups, Sister Study, (2003-2009), N=18,368

	Overall		≤High School		Some College		≥College	
	Sensitivity	Specificity	Sensitivity	Specificity	Sensitivity	Specificity	Sensitivity	Specificity
Overall	88%	97%	91%	95%	89%	97%	87%	98%
White	88%	98%	90%	96%	89%	97%	86%	99%
Black	90%	96%	93%	94%	90%	95%	91%	96%
Latina	87%	95%	93%	90%	85%	95%	84%	97%

Sensitivity is calculated as the number of ‘true positives’ (participants with obesity based on examiner measurements who were also classified as with obesity based on self-reported weight and height) divided by the sum of ‘true positives’ and ‘false negatives’ (participants with obesity based on examiner measurements but were classified as without obesity based on self-reported weight and height). Specificity is calculated as the number of ‘true negatives’ (participants without obesity based on examiner measurements who were also classified as without obesity based on self-reported weight and height) divided by the sum of ‘true negatives’ and ‘false positives’ (participants without obesity based on examiner measurements who were classified as with obesity based on self-reported weight and height).

Supplemental Table 6. Proportions, percentage agreements, kappas, and weighted kappas for categorical body mass index (BMI) among the overall population and by educational attainment, Sister Study (2003-2009), N=18,368

	BMI Category	Objectively/examiner measured proportion						
		Underweight	Recommended	Overweight	Obesity Class I	Obesity Class II	Obesity Class III	*Total*
		**Overall**						
Self-reported proportion	Underweight	0.6	0.2	0	0	0	0	0.8
	Recommended	0.4	33.3	3.8	0	0	0	37.5
	Overweight	0	2.3	26.5	3.5	0.1	0	32.4
	Obesity Class I	0	0	1.7	13.6	2.2	0.1	17.6
	Obesity Class II	0	0	0	0.9	5.3	0.9	7.1
	Obesity Class III	0	0	0	0	0.5	4.0	4.5
	Total	1.1	35.8	32.1	18.1	8.1	5.0	100
	Percentage Agreement	83%						
	Kappa (95% CI)	0.77	(0.76,	0.78)	Weighted Kappa (95% CI)	0.93	(0.93,	0.94)
		**≤High School**						
	Underweight	0.4	0.1	0	0	0	0	0.5
	Recommended	0.5	25.9	3.6	0	0	0	30.0
	Overweight	0	2.8	28.3	3.2	0.1	0	34.4
	Obesity Class I	0	0.1	2.9	15.4	2.8	0	21.2
	Obesity Class II	0	0	0	1.2	6.1	0.9	8.2
	Obesity Class III	0	0	0	0	0.5	5.2	5.7
	Total	0.9	28.8	34.8	19.9	9.4	6.1	100
	Percentage Agreement	81%						
	Kappa (95% CI)	0.75	(0.73,	0.77)	Weighted Kappa (95% CI)	0.93	(0.92,	0.93)
		**Some College**						

Underweight	0.6	0.2	0	0	0	0	0.8
Recommended	0.2	27.9	3.7	0	0	0	31.8
Overweight	0	2.5	27.4	4.0	0	0	33.9
Obesity Class I	0	0.1	1.9	15.1	2.4	0.1	19.6
Obesity Class II	0	0	0	0.9	6.4	1.1	8.4
Obesity Class III	0	0	0	0	0.6	5.0	5.6
Total	0.8	30.6	33.0	20.0	9.4	6.1	100
Percentage Agreement	82%						
Kappa (95% CI)	0.76	(0.75,	0.77)	Weighted Kappa (95% CI)	0.93	(0.93,	0.94)
				**≥College**			
Underweight	0.7	0.2	0	0	0	0	0.9
Recommended	0.5	39.5	4.0	0	0	0	44.0
Overweight	0	2	25.2	3.4	0.1	0	30.7
Obesity Class I	0	0	1.2	12.0	1.9	0.1	15.2
Obesity Class II	0	0	0	0.7	4.3	0.8	5.8
Obesity Class III	0	0	0	0	0.4	2.9	3.3
Total	1.3	41.7	30.5	16.1	6.7	3.8	100
Percentage Agreement	85%						
Kappa (95% CI)	0.78	(0.77,	0.79)	Weighted Kappa (95% CI)	0.93	(0.93,	0.94)

BMI categories are defined as follows: Underweight (BMI < 18.5 kg/m^2); Recommended (BMI 18.5 kg/m^2 to 24.9 kg/m^2); Overweight (BMI 25 kg/m^2 to 29.9 kg/m^2); Obesity Class I (BMI 30 kg/m^2 to 34.9 kg/m^2); Obesity Class II (BMI 35 kg/m^2 to 39.9 kg/m^2); and Obesity Class III (BMI ≥ 40.0 kg/m^2).

Note: Interpretations of Cohen's kappa (κ) values are no agreement (κ=0-0.20), minimal to weak agreement (0.21-0.59), moderate to strong agreement (0.60-0.90), and almost perfect agreement (≥0.90).

Supplemental Table 7. Proportions, percentage agreement, kappa, and weighted kappa for categorical body mass index (BMI) among the overall population and by educational attainment among non-Hispanic White participants, Sister Study (2003-2009), N=15,502

	BMI Category	Objectively/examiner measured proportion						
		Underweight	Recommended	Overweight	Obesity Class I	Obesity Class II	Obesity Class III	*Total*
		**All**						
Self-reported proportion	Underweight	0.7	0.2	0	0	0	0	0.9
	Recommended	0.5	35.8	3.9	0	0	0	40.2
	Overweight	0	2.2	26.3	3.4	0.1	0	32.0
	Obesity Class I	0	0	1.6	12.9	2.0	0.1	16.6
	Obesity Class II	0	0	0	0.7	5.0	0.8	6.5
	Obesity Class III	0	0	0	0	0.4	3.4	3.8
	Total	1.2	38.3	31.8	17.1	7.4	4.2	100
	Percentage Agreement	84%						
	Kappa (95% CI)	0.78	(0.77,	0.78)	Weighted Kappa (95% CI)	0.93	(0.93,	0.94)
		**≤High School**						
	Underweight	0.4	0.1	0	0	0	0	0.5
	Recommended	0.6	27.4	3.5	0	0	0	31.5
	Overweight	0	2.8	28.3	3.2	0.1	0	34.4
	Obesity Class I	0	0	2.6	15.0	2.7	0	20.3
	Obesity Class II	0	0	0	1.1	6.0	0.8	7.9
	Obesity Class III	0	0	0	0	0.4	4.8	5.2
	Total	1	30.3	34.4	19.4	9.2	5.7	100
	Percentage Agreement	82%						
	Kappa (95% CI)	0.76	(0.74,	0.78)	Weighted Kappa (95% CI)	0.93	(0.92,	0.94)
		**Some College**						

Underweight	0.7	0.2	0	0	0	0	0.9
Recommended	0.2	30.2	3.7	0	0	0	34.1
Overweight	0	2.4	27.7	3.7	0	0	33.8
Obesity Class I	0	0.1	1.8	14.7	2.2	0.1	18.9
Obesity Class II	0	0	0	0.7	6.1	0.9	7.7
Obesity Class III	0	0	0	0	0.5	4.1	4.6
Total	0.9	32.8	33.3	19.1	8.8	5.1	100
Percentage Agreement	83%						
Kappa (95% CI)	0.77	(0.76,	0.79)	Weighted Kappa (95% CI)	0.93	(0.93,	0.94)
				**≥College**			
Underweight	0.8	0.3	0	0	0	0	1.1
Recommended	0.6	42.7	4.1	0	0	0	47.4
Overweight	0	1.9	24.7	3.2	0.1	0	29.9
Obesity Class I	0	0	1.1	11.0	1.7	0.1	13.9
Obesity Class II	0	0	0	0.6	3.8	0.7	5.1
Obesity Class III	0	0	0	0	0.3	2.3	2.6
Total	1.4	44.9	29.9	14.8	5.9	3.0	100
Percentage Agreement	85%						
Kappa (95% CI)	0.78	(0.77, 0.79)		Weighted Kappa (95% CI)	0.93	(0.93,	0.94)

BMI categories are defined as follows: Underweight (BMI < 18.5 kg/m^2); Recommended (BMI 18.5 kg/m^2 to 24.9 kg/m^2); Overweight (BMI 25 kg/m^2 to 29.9 kg/m^2); Obesity Class I (BMI 30 kg/m^2 to 34.9 kg/m^2); Obesity Class II (BMI 35 kg/m^2 to 39.9 kg/m^2); and Obesity Class III (BMI ≥ 40.0 kg/m^2).

Supplemental Table 8. Proportions, percentage agreement, kappa, and weighted kappa for categorical body mass index (BMI) among the overall population and by educational attainment among Black/African American participants, Sister Study (2003-2009), N=1,857

	BMI Category	Objectively/examiner measured proportion						
		Underweight	Recommended	Overweight	Obesity Class I	Obesity Class II	Obesity Class III	*Total*
		**All**						
**Self-reported proportion**	Underweight	0.2	0.1	0	0	0	0	0.3
	Recommended	0.1	14	2.9	0.1	0	0	17.1
	Overweight	0	2.4	26.2	4.5	0.4	0	33.5
	Obesity Class I	0	0.1	1.9	20.0	3.8	0.1	25.9
	Obesity Class II	0	0	0.1	1.9	8.9	2.4	13.3
	Obesity Class III	0	0	0	0	1.2	8.8	10
	Total	0.3	16.5	31.1	26.4	14.3	11.3	100
	Percentage Agreement	78%						
	Kappa (95% CI)	0.72	(0.69,	0.74)	Weighted Kappa (95% CI)	0.92	(0.91,	0.93)
		**≤High School**						
	Underweight	0	0	0	0	0	0	0
	Recommended	0	6.9	3.8	0	0	0	10.7
	Overweight	0	3.1	23.3	3.8	0.6	0	30.8
	Obesity Class I	0	0	1.9	20.8	3.8	0	26.5
	Obesity Class II	0	0	0.6	3.1	9.4	3.1	16.2
	Obesity Class III	0	0	0	0	1.9	13.8	15.7
	Total	0	10.1	29.6	27.7	15.7	17.0	100
	Percentage Agreement	74%						
	Kappa (95% CI)	0.67	(0.58,	0.75)	Weighted Kappa (95% CI)	0.90	(0.87,	0.94)
		**Some College**						
	Underweight	0.4	0	0	0	0	0	0.4

Recommended	0	10.6	2.5	0	0	0	13.1
Overweight	0	2.7	24.7	5.6	0.3	0	33.3
Obesity Class I	0	0	2.1	20.2	3.8	0	26.1
Obesity Class II	0	0	0	2.4	10.0	2.4	14.8
Obesity Class III	0	0	0	0	1.6	10.8	12.4
Total	0.4	13.3	29.3	28.1	15.8	13.1	100
Percentage Agreement	77%						
Kappa (95% CI)	0.70	(0.66,	0.74)	Weighted Kappa (95% CI)	0.92	(0.91,	0.93)
			**≥College**				
Underweight	0.1	0.1	0	0	0	0	0.2
Recommended	0.2	17.4	2.9	0.1	0	0	20.6
Overweight	0	2.2	27.7	3.9	0.4	0	34.2
Obesity Class I	0	0.1	1.9	19.7	3.8	0.1	25.6
Obesity Class II	0	0	0	1.4	8.1	2.4	11.9
Obesity Class III	0	0	0	0	0.8	6.8	7.6
Total	0.3	19.7	32.5	25.1	13.2	9.2	100
Percentage Agreement	80%						
Kappa (95% CI)	0.73	(0.70,	0.77)	Weighted Kappa (95% CI)	0.92	(0.91,	0.93)

BMI categories are defined as follows: Underweight (BMI < 18.5 kg/m^2); Recommended (BMI 18.5 kg/m^2 to 24.9 kg/m^2); Overweight (BMI 25 kg/m^2 to 29.9 kg/m^2); Obesity Class I (BMI 30 kg/m^2 to 34.9 kg/m^2); Obesity Class II (BMI 35 kg/m^2 to 39.9 kg/m^2); and Obesity Class III (BMI ≥ 40.0 kg/m^2).

Supplemental Table 9. Proportions, percentage agreement, kappa, and weighted kappa for categorical body mass index (BMI) among the overall population and by educational attainment among Latina participants, Sister Study (2003-2009), N=1,009

	BMI Category	Objectively/examiner measured proportion						
		Underweight	Recommended	Overweight	Obesity Class I	Obesity Class II	Obesity Class III	*Total*
		**All**						
Self-reported proportion	Underweight	0.4	0.1	0	0	0	0	0.5
	Recommended	0.3	29.2	4.8	0	0	0	34.3
	Overweight	0	3	29.2	4.0	0	0	36.2
	Obesity Class I	0	0.2	3.4	12.8	2.4	0	18.8
	Obesity Class II	0	0	0	0.9	4.0	0.8	5.7
	Obesity Class III	0	0	0	0	0.1	4.6	4.7
	Total	0.7	32.5	37.4	17.6	6.4	5.4	100
	Percentage Agreement	80%						
	Kappa (95% CI)	0.72	(0.69,	0.76)	Weighted Kappa (95% CI)	0.92	(0.90,	0.93)
		**≤High School**						
	Underweight	0.5	0	0	0	0	0	0.5
	Recommended	0	21.0	4.6	0	0	0	25.6
	Overweight	0	3.2	32.4	2.3	0	0	37.9
	Obesity Class I	0	0.5	6.4	16.4	3.7	0	27.0
	Obesity Class II	0	0	0	1.4	4.1	0	5.5
	Obesity Class III	0	0	0	0	0	3.7	3.7
	Total	0.5	24.7	43.4	20.1	7.8	3.7	100
	Percentage Agreement	78%						
	Kappa (95% CI)	0.69	(0.62,	0.77)	Weighted Kappa (95% CI)	0.89	(0.85,	0.93)
		**Some College**						
	Underweight	0.3	0.3	0	0	0	0	0.6
	Recommended	0.8	27.0	5.5	0	0	0	33.3

Overweight	0	3.0	27.5	4.7	0	0	35.2
Obesity Class I	0	0.3	3.0	12.1	2.5	0	17.9
Obesity Class II	0	0	0	0.8	3.6	1.7	6.1
Obesity Class III	0	0	0	0	0	6.9	6.9
Total	1.1	30.6	36.1	17.6	6.1	8.5	100
Percentage Agreement	77%						
Kappa (95% CI)	0.69	(0.63, 0.75)		Weighted Kappa (95% CI)	0.92	(0.90, 0.94)	
			**≥College**				
Underweight	0.5	0	0	0	0	0	0.5
Recommended	0	35.4	4.2	0	0	0	39.6
Overweight	0	2.8	29.0	4.2	0	0	36.0
Obesity Class I	0	0	2.1	11.5	1.6	0	15.2
Obesity Class II	0	0	0	0.7	4.2	0.5	5.4
Obesity Class III	0	0	0	0	0.2	3.0	3.2
Total	0.5	38.2	35.4	16.4	6.1	3.5	100
Percentage Agreement	84%						
Kappa (95% CI)	0.76	(0.71, 0.81)		Weighted Kappa (95% CI)	0.93	(0.91, 0.95)	

BMI categories are defined as follows: Underweight (BMI < 18.5 kg/m^2); Recommended (BMI 18.5 kg/m^2 to 24.9 kg/m^2); Overweight (BMI 25 kg/m^2 to 29.9 kg/m^2); Obesity Class I (BMI 30 kg/m^2 to 34.9 kg/m^2); Obesity Class II (BMI 35 kg/m^2 to 39.9 kg/m^2); and Obesity Class III (BMI ≥ 40.0 kg/m^2).

Supplemental Table 10. Bland-Altman plot statistics: Bias (mean of differences between self-report and objective measures) and limits of agreement by educational attainment within racial and ethnic groups among participants who completed self-reports either prior to or after objective measurements, Sister Study (2003-2009), N=46,618

	Mean of Differences	SD of Differences	(LLoA, ULoA)	Mean of Differences	SD of Differences	(LLoA, ULoA)	Mean of Differences	SD of Differences	(LLoA, ULoA)
	White			Black			Latina		
**Weight (kg)**									
≤High School	-0.71	2.75	(-6.09, 4.68)	-0.85	4.05	(-8.79, 7.10)	-0.11	3.03	(-6.05, 5.83)
Some College	-0.90	2.80	(-6.38, 4.59)	-1.10	3.92	(-8.79, 6.59)	-0.71	3.20	(-6.97, 5.56)
≥College	-0.85	2.46	(-5.67, 3.97)	-1.31	3.58	(-8.32, 5.70)	-0.78	2.84	(-6.35, 4.79)
**Height (cm)**									
≤High School	-0.25	2.22	(-4.61, 4.11)	-0.43	2.94	(-6.20, 5.35)	0.05	3.53	(-6.88, 6.97)
Some College	-0.23	2.03	(-4.21, 3.75)	-0.41	2.69	(-5.69, 4.86)	-0.25	3.26	(-6.64, 6.14)
≥College	-0.24	1.85	(-3.87, 3.38)	-0.39	2.51	(-5.32, 4.53)	-0.08	2.52	(-5.02, 4.86)
**BMI (kg/m^2)**									
≤High School	-0.18	1.29	(-2.70, 2.35)	-0.16	1.93	(-3.95, 3.63)	-0.06	1.72	(-3.44, 3.32)
Some College	-0.26	1.26	(-2.72, 2.21)	-0.26	1.72	(-3.62, 3.11)	-0.20	1.64	(-3.41, 3.01)
≥College	-0.24	1.09	(-2.38, 1.91)	-0.33	1.59	(-3.45, 2.79)	-0.27	1.44	(-3.09, 2.54)

Abbreviations: SD (standard deviation); LLoA (lower limit of agreement); ULoA (upper limit of agreement)

Note: An estimated 95% of differences are within the limits of agreement.

Green shading indicates ‘good’ agreement, defined as a width of the limits of agreement that is within one standard deviation of the objectively measured mean value.

Yellow shading indicates ‘fair’ agreement, defined as a width of the limits of agreement that equals greater than one but less than or equal to two standard deviations of the objectively measured mean value.

Red shading indicates ‘poor’ agreement, defined as a width of the limits of agreement that equals greater than two standard deviations of the objectively measured mean value.

Supplemental Table 11. Mean differences between self-reported and objectively/examiner measured weight, height, and body mass index, overall and by educational attainment among participants who completed self-reports either prior to or after objective measurements within the overall population and within racial and ethnic groups, Sister Study (2003-2009), N=46,618

	Overall							≤High School							Some College							≥College						
	Self-Reported		Objectively-Measured					Self-Reported		Objectively-Measured					Self-Reported		Objectively-Measured					Self-Reported		Objectively-Measured				
	M	(SD)	M	(SD)	MD	(95% CI)		M	(SD)	M	(SD)	MD	(95% CI)		M	(SD)	M	(SD)	MD	(95% CI)		M	(SD)	M	(SD)	MD	(95% CI)	
**Weight (kg)**																												
Overall	73.9	(16.2)	74.8	(16.7)	**-0.86**	**(-0.89,**	**-0.84)**	75.7	(16.4)	76.4	(16.8)	**-0.67**	**(-0.74,**	**-0.60)**	75.8	(16.7)	76.7	(17.2)	**-0.91**	**(-0.95,**	**-0.86)**	72.1	(15.6)	73.0	(16.2)	**-0.89**	**(-0.92,**	**-0.86)**
White	73.1	(15.9)	74.0	(16.4)	**-0.84**	**(-0.87,**	**-0.82)**	75.3	(16.1)	76.0	(16.5)	**-0.71**	**(-0.78,**	**-0.64)**	75.1	(16.4)	76.0	(16.9)	**-0.90**	**(-0.94,**	**-0.85)**	71.1	(15.1)	72.0	(15.7)	**-0.85**	**(-0.88,**	**-0.81)**
Black	82.9	(17.6)	84.1	(18.2)	**-1.19**	**(-1.30,**	**-1.07)**	86.1	(18.3)	86.9	(18.6)	**-0.85**	**(-1.24,**	**-0.46)**	84.2	(17.7)	85.3	(18.3)	**-1.10**	**(-1.30,**	**-0.90)**	81.3	(17.3)	82.7	(18.0)	**-1.31**	**(-1.46,**	**-1.16)**
Latina	71.9	(14.8)	72.5	(15.3)	**-0.60**	**(-0.72,**	**-0.47)**	72.8	(14.4)	72.9	(14.9)	-0.11	(-0.36,	0.15)	72.1	(15.0)	72.8	(15.8)	**-0.71**	**(-0.93,**	**-0.48)**	71.4	(14.8)	72.1	(15.3)	**-0.78**	**(-0.96,**	**-0.60)**
**Height (cm)**																												
Overall	164.0	(6.5)	164.3	(6.4)	**-0.25**	**(-0.27,**	**-0.23)**	162.8	(6.4)	163.1	(6.3)	**-0.24**	**(-0.29,**	**-0.18)**	163.8	(6.5)	164.1	(6.4)	**-0.25**	**(-0.28,**	**-0.21)**	164.5	(6.5)	164.8	(6.4)	**-0.25**	**(-0.28,**	**-0.23)**
White	164.2	(6.5)	164.5	(6.3)	**-0.24**	**(-0.26,**	**-0.22)**	163.1	(6.3)	163.4	(6.1)	**-0.25**	**(-0.31,**	**-0.19)**	164.1	(6.4)	164.3	(6.3)	**-0.23**	**(-0.26,**	**-0.20)**	164.7	(6.5)	164.9	(6.3)	**-0.24**	**(-0.27,**	**-0.22)**
Black	164.1	(6.7)	164.5	(6.5)	**-0.40**	**(-0.48,**	**-0.32)**	163.1	(6.6)	163.6	(6.4)	**-0.43**	**(-0.71,**	**-0.14)**	163.8	(6.7)	164.2	(6.5)	**-0.41**	**(-0.55,**	**-0.28)**	164.5	(6.7)	164.8	(6.5)	**-0.39**	**(-0.50,**	**-0.29)**
Latina	160.2	(6.4)	160.3	(6.2)	-0.11	(-0.23,	0.02)	159.1	(6.3)	159.1	(6.0)	0.05	(-0.25,	0.34)	160.1	(6.7)	160.3	(6.5)	**-0.25**	**(-0.48,**	**-0.02)**	160.9	(6.1)	161.0	(6.1)	-0.08	(-0.24,	0.08)
**BMI (kg/m^2)**																												
Overall	27.5	(5.8)	27.8	(6.0)	**-0.24**	**(-0.25,**	**-0.23)**	28.6	(5.9)	28.8	(6.1)	**-0.17**	**(-0.20,**	**-0.13)**	28.3	(6.0)	28.6	(6.2)	**-0.25**	**(-0.27,**	**-0.23)**	26.7	(5.6)	26.9	(5.8)	**-0.25**	**(-0.26,**	**-0.23)**
White	27.2	(5.7)	27.4	(5.9)	**-0.23**	**(-0.25,**	**-0.22)**	28.4	(5.9)	28.5	(6.0)	**-0.18**	**(-0.21,**	**-0.14)**	28.0	(5.8)	28.2	(6.0)	**-0.26**	**(-0.28,**	**-0.24)**	26.3	(5.4)	26.5	(5.6)	**-0.24**	**(-0.25,**	**-0.22)**
Black	30.8	(6.3)	31.1	(6.5)	**-0.29**	**(-0.34,**	**-0.24)**	32.4	(6.6)	32.6	(6.7)	-0.16	(-0.34,	0.03)	31.5	(6.3)	31.7	(6.5)	**-0.26**	**(-0.34,**	**-0.17)**	30.1	(6.1)	30.5	(6.3)	**-0.33**	**(-0.40,**	**-0.27)**
Latina	28.1	(5.4)	28.3	(5.7)	**-0.20**	**(-0.26,**	**-0.13)**	28.7	(5.3)	28.8	(5.4)	-0.06	(-0.21,	0.08)	28.2	(5.6)	28.4	(5.9)	**-0.20**	**(-0.32,**	**-0.09)**	27.6	(5.4)	27.9	(5.6)	**-0.27**	**(-0.36,**	**-0.18)**

Abbreviations: M (mean); SD (standard deviation); MD (mean difference); kg (kilograms); cm (centimeters); kg/m^2 (kilograms/meters^2)
Boldface indicates mean differences with a p-value <0.05.

Supplemental Table 12. Proportions, percentage agreement, kappa, and weighted kappa for categorical body mass index (BMI) among participants who completed self-reports either prior to or after objective measurements, overall and by race and ethnicity, Sister Study (2003-2009), N=46,618

Self-reported proportion	Objectively-measured proportion						
	Underweight	Normal	Overweight	Obesity Class I	Obesity Class II	Obesity Class III	Total
**All**							
Underweight	0.6	0.2	0	0	0	0	0.8
Recommended	0.4	34.6	3.4	0	0	0	38.4
Overweight	0	2.1	27.2	3.1	0	0	32.4
Obesity Class I	0	0	1.5	13.5	1.9	0.1	17.0
Obesity Class II	0	0	0	0.8	5.4	0.9	7.1
Obesity Class III	0	0	0	0	0.4	3.9	4.3
Total	1.0	36.9	32.1	17.4	7.7	4.8	100
Percentage Agreement	85%						
Kappa (95% CI)	0.79	(0.79,	0.80)	Weighted Kappa (95% CI)	0.94	(0.94,	0.94)
**White**							
Underweight	0.7	0.2	0	0	0	0	0.9
Recommended	0.5	37.1	3.4	0	0	0	41.0
Overweight	0	2.1	27	3	0	0	32.1
Obesity Class I	0	0	1.4	12.8	1.7	0.1	16
Obesity Class II	0	0	0	0.6	4.9	0.7	6.2
Obesity Class III	0	0	0	0	0.3	3.4	3.7
Total	1.1	39.4	31.8	16.4	7.0	4.1	100
Percentage Agreement	86%						
Kappa (95% CI)	0.80	(0.79,	0.80)	Weighted Kappa (95% CI)	0.94	(0.94,	0.94)

				**Black**			
Underweight	0.2	0	0	0	0	0	0.2
Recommended	0.1	14.1	2.3	0	0	0	16.5
Overweight	0	2.1	27.6	4.1	0.2	0	34.0
Obesity Class I	0	0	2.1	19.7	3.5	0.1	25.4
Obesity Class II	0	0	0	2.1	9.4	2.2	13.7
Obesity Class III	0	0	0	0	1.1	8.8	9.9
Total	0.3	16.3	32.1	25.8	14.3	11.1	100
Percentage Agreement	80%						
Kappa (95% CI)	0.74	(0.72, 0.75)		Weighted Kappa (95% CI)	0.93	(0.92, 0.93)	
				**Hispanic/Latina**			
Underweight	0.4	0.1	0	0	0	0	0.5
Recommended	0.3	27.9	4.2	0	0	0	32.4
Overweight	0	2.5	30.4	3.8	0	0	36.7
Obesity Class I	0	0.2	2.8	14.3	2	0.1	19.4
Obesity Class II	0	0	0.1	1	5.1	0.8	7.0
Obesity Class III	0	0	0	0	0.3	3.7	4.0
Total	0.7	30.6	37.5	19.2	7.5	4.6	100
Percentage Agreement	82%						
Kappa (95% CI)	0.75	(0.73, 0.77)		Weighted Kappa (95% CI)	0.92	(0.91, 0.93)	

BMI categories are defined as follows: Underweight (BMI < 18.5 kg/m2); Recommended (BMI 18.5 kg/m2 to 24.9 kg/m2); Overweight (BMI 25 kg/m2 to 29.9 kg/m2); Obesity Class I (BMI 30 kg/m2 to 34.9 kg/m2); Obesity Class II (BMI 35 kg/m2 to 39.9 kg/m2); and Obesity Class III (BMI ≥ 40.0 kg/m2).

Supplemental Table 13. Proportions, percentage agreement, kappa, and weighted kappa for categorical body mass index (BMI) among participants who completed self-reports either prior to or after objective measurements, overall and by educational attainment, Sister Study (2003-2009), N=46,618

Self-reported proportion

	Objectively-measured proportion						
	Underweight	Normal	Overweight	Obesity Class I	Obesity Class II	Obesity Class III	Total
Self-reported proportion				**All**			
Underweight	0.6	0.2	0	0	0	0	0.8
Recommended	0.4	34.6	3.4	0	0	0	38.4
Overweight	0	2.1	27.2	3.1	0	0	32.4
Obesity Class I	0	0	1.5	13.5	1.9	0.1	17
Obesity Class II	0	0	0	0.8	5.4	0.9	7.1
Obesity Class III	0	0	0	0	0.4	3.9	4.3
Total	1	36.9	32.1	17.4	7.7	4.8	100
Percentage Agreement	85%						
Kappa (95% CI)	0.79	(0.79,	0.80)	Weighted Kappa (95% CI)	0.94	(0.94,	0.94)
				**≤High School**			
Underweight	0.4	0.1	0	0	0	0	0.5
Recommended	0.4	26.2	3.1	0	0	0	29.7
Overweight	0	2.6	29.3	3.1	0.1	0	35.1
Obesity Class I	0	0.1	2.3	16	2.2	0	20.6
Obesity Class II	0	0	0.1	1.2	6.4	1	8.7
Obesity Class III	0	0	0	0	0.6	4.9	5.5
Total	0.8	28.9	34.7	20.4	9.2	6.0	100
Percentage Agreement	83%						
Kappa (95% CI)	0.77	(0.76,	0.78)	Weighted Kappa	0.93	(0.93,	0.94)

				(95% CI)			
				**Some College**			
Underweight	0.6	0.2	0	0	0	0	0.8
Recommended	0.4	28.9	3.3	0	0	0	32.6
Overweight	0	2.1	28	3.5	0	0	33.6
Obesity Class I	0	0.1	1.7	15.2	2.2	0.1	19.3
Obesity Class II	0	0	0	0.9	6.6	1	8.5
Obesity Class III	0	0	0	0	0.5	4.8	5.3
Total	0.9	31.3	33.0	19.6	9.2	5.9	100
Percentage Agreement	84%						
Kappa (95% CI)	0.78	(0.78, 0.79)		Weighted Kappa (95% CI)	0.94	(0.94, 0.94)	
				**≥College**			
Underweight	0.7	0.2	0	0	0	0	0.9
Recommended	0.5	40.9	3.5	0	0	0	44.9
Overweight	0	1.9	26.1	2.9	0.1	0	31.0
Obesity Class I	0	0	1.2	11.5	1.6	0.1	14.4
Obesity Class II	0	0	0	0.6	4.2	0.7	5.5
Obesity Class III	0	0	0	0	0.3	2.9	3.2
Total	1.2	43.1	30.8	15.1	6.2	3.7	100
Percentage Agreement	86%						
Kappa (95% CI)	0.80	(0.79, 0.81)		Weighted Kappa (95% CI)	0.94	(0.94, 0.94)	

BMI categories are defined as follows: Underweight (BMI < 18.5 kg/m2); Recommended (BMI 18.5 kg/m2 to 24.9 kg/m2); Overweight (BMI 25 kg/m2 to 29.9 kg/m2); Obesity Class I (BMI 30 kg/m2 to 34.9 kg/m2); Obesity Class II (BMI 35 kg/m2 to 39.9 kg/m2); and Obesity Class III (BMI ≥ 40.0 kg/m2).

Supplemental Table 14. Proportions, percentage agreement, kappa, and weighted kappa for categorical body mass index (BMI) among non-Hispanic White participants who completed self-reports either prior to or after objective measurements, overall and by educational attainment, Sister Study (2003-2009), N=40,145

Self-reported proportion

	Objectively-measured proportion						
	Underweight	Normal	Overweight	Obesity Class I	Obesity Class II	Obesity Class III	Total
Self-reported proportion			**All**				
Underweight	0.7	0.2	0	0	0	0	0.9
Recommended	0.5	37.1	3.4	0	0	0	41
Overweight	0	2.1	27	3	0	0	32.1
Obesity Class I	0	0	1.4	12.8	1.7	0.1	16
Obesity Class II	0	0	0	0.6	4.9	0.7	6.2
Obesity Class III	0	0	0	0	0.3	3.4	3.7
Total	1.1	39.4	31.8	16.4	7.0	4.1	100
Percentage Agreement	85%						
Kappa (95% CI)	0.80	(0.79,	0.80)	Weighted Kappa (95% CI)	0.94	(0.94,	0.94)
			**≤High School**				
Underweight	0.4	0.1	0	0	0	0	0.5
Recommended	0.4	27.9	3.2	0	0	0	31.5
Overweight	0	2.5	29.3	3.1	0	0	34.9
Obesity Class I	0	0	2.1	15.7	2.1	0	19.9
Obesity Class II	0	0	0	1	6.2	0.9	8.1
Obesity Class III	0	0	0	0	0.5	4.5	5.0
Total	0.9	30.5	34.6	19.7	8.7	5.5	100
Percentage Agreement	84%						
Kappa (95% CI)	0.78	(0.77,	0.79)	Weighted Kappa	0.94	(0.93,	0.94)

				(95% CI)			
**Some College**							
Underweight	0.6	0.2	0	0	0	0	0.8
Recommended	0.4	31	3.4	0	0	0	34.8
Overweight	0	2.2	28.1	3.4	0	0	33.7
Obesity Class I	0	0	1.6	14.6	2	0.1	18.3
Obesity Class II	0	0	0	0.7	6.2	0.8	7.7
Obesity Class III	0	0	0	0	0.4	4.3	4.7
Total	1.0	33.3	33.1	18.7	8.6	5.2	100
Percentage Agreement	85%						
Kappa (95% CI)	0.79	(0.78,	0.80)	Weighted Kappa (95% CI)	0.94	(0.94,	0.94)
**≥College**							
Underweight	0.8	0.2	0	0	0	0	1.0
Recommended	0.5	44	3.5	0	0	0	48.0
Overweight	0	1.9	25.5	2.7	0	0	30.1
Obesity Class I	0	0	1.1	10.7	1.4	0	13.2
Obesity Class II	0	0	0	0.5	3.8	0.6	4.9
Obesity Class III	0	0	0	0	0.3	2.4	2.7
Total	1.3	46.1	30.1	13.9	5.5	3.1	100
Percentage Agreement	87%						
Kappa (95% CI)	0.81	(0.80,	0.81)	Weighted Kappa (95% CI)	0.94	(0.94,	0.94)

BMI categories are defined as follows: Underweight (BMI < 18.5 kg/m2); Recommended (BMI 18.5 kg/m2 to 24.9 kg/m2); Overweight (BMI 25 kg/m2 to 29.9 kg/m2); Obesity Class I (BMI 30 kg/m2 to 34.9 kg/m2); Obesity Class II (BMI 35 kg/m2 to 39.9 kg/m2); and Obesity Class III (BMI ≥ 40.0 kg/m2).

Supplemental Table 15. Proportions, percentage agreement, kappa, and weighted kappa for categorical body mass index (BMI) among non-Hispanic Black/African American participants who completed self-reports either prior to or after objective measurements, overall and by educational attainment, Sister Study (2003-2009), N=4,156

	Objectively-measured proportion							
Self-reported proportion		Underweight	Normal	Overweight	Obesity Class I	Obesity Class II	Obesity Class III	Total
	Self-reported proportion	**All**						
	Underweight	0.2	0	0	0	0	0	0.2
	Recommended	0.1	14.1	2.3	0	0	0	16.5
	Overweight	0	2.1	27.6	4.1	0.2	0	34.0
	Obesity Class I	0	0	2.1	19.7	3.5	0.1	25.4
	Obesity Class II	0	0	0	2.1	9.4	2.2	13.7
	Obesity Class III	0	0	0	0	1.1	8.8	9.9
	Total	0.3	16.3	32.1	25.8	14.3	11.1	100
	Percentage Agreement	80%						
	Kappa (95% CI)	0.74	(0.72, 0.75)		Weighted Kappa (95% CI)	0.93	(0.92, 0.93)	
		**≤High School**						
	Underweight	0.2	0	0	0	0	0	0.2
	Recommended	0.2	7.2	2.2	0.2	0	0	9.8
	Overweight	0	3.4	23.5	4.6	0.2	0	31.7
	Obesity Class I	0	0	1.9	19.2	3.8	0.2	25.1
	Obesity Class II	0	0	0.2	3.1	11.5	3.4	18.2
	Obesity Class III	0	0	0	0	2.6	12.2	14.8
	Total	0.5	10.6	27.8	27.1	18.2	15.8	100
	Percentage Agreement	74%						
	Kappa (95% CI)	0.66	(0.61, 0.72)		Weighted Kappa	0.91	(0.89, 0.93)	

				(95% CI)			
**Some College**							
Underweight	0.3	0.1	0	0	0	0	0.4
Recommended	0.1	11.5	1.9	0	0	0	13.5
Overweight	0	2	26.2	4.1	0.1	0	32.4
Obesity Class I	0	0	2.5	21.5	3.4	0.2	27.6
Obesity Class II	0	0	0	2.1	10.5	2.1	14.7
Obesity Class III	0	0	0	0	1.2	10.2	11.4
Total	0.4	13.5	30.6	27.7	15.3	12.5	100
Percentage Agreement	80%						
Kappa (95% CI)	0.74	(0.72, 0.77)		Weighted Kappa (95% CI)	0.93	(0.92, 0.94)	
**≥College**							
Underweight	0.1	0	0	0	0	0	0.1
Recommended	0.2	17.2	2.5	0	0	0	19.9
Overweight	0	1.9	29.4	4	0.3	0	35.6
Obesity Class I	0	0	1.9	18.5	3.6	0	24.0
Obesity Class II	0	0	0	1.8	8.3	2	12.1
Obesity Class III	0	0	0	0	0.7	7.3	8.0
Total	0.3	19.2	33.9	24.4	12.9	9.3	100
Percentage Agreement	81%						
Kappa (95% CI)	0.75	(0.73, 0.77)		Weighted Kappa (95% CI)	0.93	(0.92, 0.94)	

BMI categories are defined as follows: Underweight (BMI < 18.5 kg/m2); Recommended (BMI 18.5 kg/m2 to 24.9 kg/m2); Overweight (BMI 25 kg/m2 to 29.9 kg/m2); Obesity Class I (BMI 30 kg/m2 to 34.9 kg/m2); Obesity Class II (BMI 35 kg/m2 to 39.9 kg/m2); and Obesity Class III (BMI ≥ 40.0 kg/m2).

Supplemental Table 16. Proportions, percentage agreement, kappa, and weighted kappa for categorical body mass index (BMI) among Hispanic/Latina participants who completed self-reports either prior to or after objective measurements, overall and by educational attainment, Sister Study (2003-2009), N=2,317

Self-reported proportion	Objectively-measured proportion: Underweight	Normal	Overweight	Obesity Class I	Obesity Class II	Obesity Class III	Total
**All**							
Underweight	0.4	0.1	0	0	0	0	0.5
Recommended	0.3	27.9	4.2	0	0	0	32.4
Overweight	0	2.5	30.4	3.8	0	0	36.7
Obesity Class I	0	0.2	2.8	14.3	2	0.1	19.4
Obesity Class II	0	0	0.1	1	5.1	0.8	7.0
Obesity Class III	0	0	0	0	0.3	3.7	4.0
Total	0.7	30.6	37.5	19.2	7.5	4.6	100
Percentage Agreement	82%						
Kappa (95% CI)	0.75	(0.73,	0.77)	Weighted Kappa (95% CI)	0.92	(0.91,	0.93)
**≤High School**							
Underweight	0.4	0	0	0	0	0	0.4
Recommended	0	21.5	3.1	0	0	0	24.6
Overweight	0	2.9	33.4	2.9	0	0	39.2
Obesity Class I	0	0.2	4	18	2.6	0	24.8
Obesity Class II	0	0	0.4	1.3	4.8	0.4	6.9
Obesity Class III	0	0	0	0	0.7	3.5	4.2
Total	0.4	24.6	40.9	22.2	8.1	3.9	100
Percentage Agreement	81%						
Kappa (95% CI)	0.74	(0.70,	0.79)	Weighted Kappa	0.91	(0.89,	0.93)

				(95% CI)			
				**Some College**			
Underweight	0.4	0.3	0	0	0	0	0.7
Recommended	0.5	27.8	4.3	0	0	0	32.6
Overweight	0	2	29.5	4.1	0.1	0	35.7
Obesity Class I	0	0.4	2.7	13.3	2	0.3	18.7
Obesity Class II	0	0	0	1	5.2	1.4	7.6
Obesity Class III	0	0	0	0	0.1	4.6	4.7
Total	0.9	30.5	36.5	18.4	7.5	6.3	100
Percentage Agreement	81%						
Kappa (95% CI)	0.74	(0.70,	0.77)	Weighted Kappa (95% CI)	0.92	(0.90,	0.93)
				**≥College**			
Underweight	0.4	0	0	0	0	0	0.4
Recommended	0.3	31.5	4.7	0	0	0	36.5
Overweight	0	2.5	29.5	4	0	0	36.0
Obesity Class I	0	0	2.1	13.2	1.6	0.1	17.0
Obesity Class II	0	0	0.1	0.9	5.3	0.5	6.8
Obesity Class III	0	0	0	0	0.2	3.1	3.3
Total	0.7	34.0	36.3	18.1	7.1	3.7	100
Percentage Agreement	83%						
Kappa (95% CI)	0.76	(0.73,	0.79)	Weighted Kappa (95% CI)	0.92	(0.91,	0.94)

BMI categories are defined as follows: Underweight (BMI < 18.5 kg/m2); Recommended (BMI 18.5 kg/m2 to 24.9 kg/m2); Overweight (BMI 25 kg/m2 to 29.9 kg/m2); Obesity Class I (BMI 30 kg/m2 to 34.9 kg/m2); Obesity Class II (BMI 35 kg/m2 to 39.9 kg/m2); and Obesity Class III (BMI ≥ 40.0 kg/m2).
